# Intravenous iron versus blood transfusion for postpartum anemia: a systematic review and meta-analysis

**DOI:** 10.1186/s13643-023-02400-4

**Published:** 2024-01-02

**Authors:** E. Caljé, K. M. Groom, L. Dixon, J. Marriott, R. Foon, C. Oyston, F. H. Bloomfield, V. Jordan

**Affiliations:** 1 Liggins Institute, University of Auckland, Auckland, New Zealand; 2 National Women’s Health, Auckland City Hospital, Auckland, New Zealand; 3 New Zealand College of Midwives, Christchurch, New Zealand; 4 Department of Obstetrics and Gynaecology, Faculty of Medicine and Health Sciences, The University of Auckland, Auckland, New Zealand; 5 Waikato Hospital, Hamilton, New Zealand; 6 Middlemore Hospital, Auckland, New Zealand

**Keywords:** Anemia, Erythrocyte transfusion, Iron deficiency, Ferric compounds, Hematinics, Fatigue, Puerperal disorders, Adverse drug reaction, Intravenous infusion, Iron

## Abstract

**Background:**

Intravenous iron (IV-iron) is used as an alternative to, or alongside, red blood cell transfusion (RBC-T) to treat more severe postpartum anemia (PPA), although optimal treatment options remain unclear. No previous systematic reviews have examined IV-iron and RBC-T, including patient-reported outcomes and hematological responses.

**Methods:**

A systematic review and meta-analysis of randomized trials comparing IV-iron and RBC-T with each other, oral iron, no treatment, and placebo for the treatment of PPA. Key inclusion criteria were PPA (hemoglobin < 12 g/dL) and IV-iron or RBC-T as interventions. Key exclusion criteria were antenatal IV-iron or RBC-T. Fatigue was the primary outcome. Secondary outcomes included hemoglobin and ferritin concentrations, and adverse events. From 27th August 2020 to 26th September 2022, databases, registries, and hand searches identified studies. A fixed-effect meta-analysis was undertaken using RevMan (5.4) software. The quality of the studies and the evidence was assessed using the Cochrane Risk of Bias table, and Grading of Recommendations, Assessment, Development, and Evaluation. This review is registered with the Prospective Register of Systematic Reviews (CRD42020201115).

**Results:**

Twenty studies and 4196 participants were included: 1834 assigned IV-iron, 1771 assigned oral iron, 330 assigned RBC-T, and 261 assigned non-intervention. Six studies reported the primary outcome of fatigue (1251 participants). Only studies of IV-iron vs. oral iron (15 studies) were available for meta-analysis. Of these, three reported on fatigue using different scales; two were available for meta-analysis. There was a significant reduction in fatigue with IV-iron compared to oral iron (standardized mean difference − 0.40, 95% confidence interval (CI) − 0.62, − 0.18, *I*^2^ = 0%). The direction of effect also favored IV-iron for hemoglobin (mean difference (MD) 0.54 g/dL, 95% confidence interval (CI) 0.47, 0.61, *I*^2^ = 91%), ferritin, (MD 58.07 mcg/L, 95% CI 55.74, 60.41, *I*^2^ = 99%), and total adverse events (risk-ratio 0.63, 95% CI 0.52, 0.77, *I*^2^ = 84%). The overall quality of the evidence was low-moderate.

**Discussion:**

For all outcomes, the evidence for RBC-T, compared to IV-iron, non-intervention, or dose effects of RBC-T is very limited. Further research is needed to determine whether RBC-T or IV-iron for the treatment of PPA is superior for fatigue and hematological outcomes.

**Supplementary Information:**

The online version contains supplementary material available at 10.1186/s13643-023-02400-4.

## Introduction

Postpartum anemia (PPA), a low concentration of hemoglobin (Hb) after childbirth, results in reduced oxygen-carrying capacity that may mean it is more difficult for mothers to meet the physiological demands of recovery from birth and support for their newborn. PPA is most commonly caused by iron-deficiency anemia before birth, and/or excessive bleeding at birth [1–3].

PPA is strongly associated with increased morbidity and mortality [4, 5]. Women with postpartum anemia are also more likely to experience fatigue, altered cognition, and depressive symptoms which may affect interactions with their infants, impacting infant behavior and development [6]. Without treatment for PPA, the resumption of everyday activities is more difficult for women after birth [7]. Although PPA is common, prevalence data are limited [6]. Estimates suggest that a third of all postpartum women have PPA [8]. Even in high-income countries, PPA contributes significantly to the global burden of anemia [4, 5].

The main treatment options for PPA are oral iron, intravenous iron (IV-iron), and red blood cell transfusion (RBC-T). When PPA is more severe, the treatment choice is often between IV-iron and RBC-T. Current guidelines [9] and patient blood management strategies [10, 11] recommend IV-iron as an alternative to RBC-T for hemodynamically stable postpartum women who are not actively bleeding.

A 2015 Cochrane review [3] with primary outcomes of fatigue and maternal mortality included only one trial [12] with RBC-T as an intervention for PPA. More recently published trials that include RBC-T were not included in recent systematic reviews that focussed on IV-iron and oral iron treatments [13, 14]. Therefore, it is timely to re-examine and update the evidence to guide clinical practice and identify evidence gaps. We undertook a systematic review of all completed randomized trials to assess the effects of IV-iron and RBC-T for PPA with the assessment of patient-reported outcomes, hematological response, and safety. Fatigue was selected as the primary outcome because there is growing recognition of the complex relationship between postpartum fatigue, depression [15–18] and anemia [19–24]. Fatigue is also correlated with hemoglobin levels [25].

## Methods

A systematic review and meta-analysis of randomized trials comparing IV-iron and RBC-T with each other; or IV-iron or RBC-T with oral iron, no treatment, or placebo for the treatment for women with PPA. This systematic review was undertaken and reported following methods in the: Cochrane Handbook for Systematic Reviews [26]; the Preferred Reporting Items for Systematic Reviews and Meta-Analysis (PRISMA, including the checklist (Additional File 2 Appendix 2)) [27]; the Grading of Recommendations, Assessment, Development and Evaluation (GRADE) [28]; and registered with PROSPERO on 23rd September 2020 (CRD42020201115).

### Eligibility criteria

Randomized trials included were those that assessed IV-iron and/or RBC-T as treatment interventions for PPA, defined broadly as postpartum Hb < 12 g/dL up to 6 weeks after birth. Eligible studies included completed randomized, or cluster-randomized trials, published and unpublished. Types of studies excluded were non-randomized, quasi-experimental, cohort and cross-over design studies, non-English publications, reviews, comments, case reports, and animal studies. Studies were excluded if IV-iron or RBC-T were not trial interventions, if IV-iron or RBC-T were given antenatally, or if erythropoietin or high molecular weight iron dextran were study interventions. There were no exclusion criteria for outcomes.

### Information sources and search strategy

Literature searches were run from the database inceptions to 26th September 2022 in the following databases and registries for randomized trials comparing the efficacy of IV-iron and/or RBC-T with each other, oral iron or placebo: MEDLINE (Ovid), EMBASE (Ovid), Scopus, Cumulative Index to Nursing and Allied Health Literature, Web of Science Core Collection, Cochrane Central Register of Controlled Trials, Latin-American and Caribbean Health Science Literature database, Australia and New Zealand Clinical Trials Registry, and ClinicalTrials.gov. There was no date limitation for the included studies. Hand-searching was also undertaken from citation searches.

The literature search included the following Medical Subject Headings and keywords: adverse effects, anemia, iron deficiency, erythrocyte transfusion, ferric compounds, ferrous compounds, hematinics, intravenous injections, iron, postpartum period, puerperal disorders, and randomized controlled trials. Full details of the search criteria for the MEDLINE database are outlined in Additional file Appendix 1.

### Study screening and data extraction

Identified studies were imported into Covidence software (version 1.0, Veritas Health Innovation Ltd) to screen for eligibility and exclusion criteria. Independent study selection and inclusion were undertaken by two reviewers (EC and LD). Discrepancies were resolved by consensus, or with a third reviewer (KG or VJ). One reviewer extracted the data using a customized Microsoft Excel data extraction tool, after piloting the tool. A second reviewer from the investigator team (LD, JM, or RF) randomly selected and independently extracted the data for eleven (11/20) of the studies. Where data were presented only in a graphical format, the data were visually extracted from the graphs and independently checked with a second reviewer. Any concerns around study selection, missing results, data extraction, and inclusion in the meta-analysis were reviewed by the senior investigator (VJ). When clarification was required, authors were contacted for information on the data and quality assessment processes.

Extracted data included the following: bias assessment, location and year of study, duration of study period and recruitment, methodology, inclusion and exclusion criteria, demographic data, number of participants and dropouts, iron formulations including dosing regimens, baseline Hb and ferritin concentrations, and information on various measures of outcomes. Outcome data measurements included fatigue scores, hemoglobin and ferritin concentrations, symptoms of anemia, drug adverse effects, breastfeeding rates, depression scores, and other patient-reported health-related quality-of-life outcomes. Data were entered into Review Manager (RevMan 5.4, 2020–http://tech.cochrane.org/revman) software and checked for accuracy by the investigator team.

### Outcomes

The primary outcome was fatigue, measured by any dichotomous patient reporting, unidimensional, or multidimensional scales. The main secondary outcome was hemoglobin, as an objective measure for assessing biological response to iron interventions [13]. Hemoglobin was measured as concentration and as clinically relevant responses, defined as an increase in Hb ≥ 2.0 g/dL from baseline [29] or a final Hb > 12 g/dL [13]. Other secondary outcomes included ferritin (concentration and change from baseline), adverse effects, breastfeeding, alleviation of anemia symptoms, psychological well-being measured by the Edinburgh Postnatal Depression Score (EPDS) [30] and other HRQoL measures such as Medical Outcome Study 36-Item Short-Form Health Survey (SF-36) [31].

### Study quality assessment

The quality of the included studies was assessed with the Cochrane Collaboration Tool for evaluating the risk of bias (ROB1) [26]. Two reviewers independently evaluated the methodological quality of the studies against the specific criteria and study domains [26]: random sequence generation, allocation concealment, blinding of participants and personnel, blinding of outcome assessment, incomplete outcome data, selective reporting, and other potential sources of bias. Bias for each of the criteria was reported as high, low, or unclear risk of bias. An ‘unclear’ response indicated uncertainty about the trial process and/or no information. The magnitude of any domain bias and the impact on findings was evaluated using the Cochrane Handbook [26].

After meta-analysis, the overall quality of the evidence and completeness of pre-specified outcomes (of fatigue, hemoglobin, ferritin, and adverse effects) was assessed by the reviewers, according to the GRADE categories of study design, risk of bias, imprecision, inconsistency, indirectness, and magnitude of effect [28]. Disagreements were resolved by consensus, or by discussion with the senior investigator (VJ). The overall quality of the evidence was presented in the summary of findings table.

### Data synthesis

Meta-analyses were performed with the Cochrane Review Manager software (RevMan 5.4) using a fixed-effects model. For all continuous data, the mean differences with 95% confidence intervals (CIs) were calculated. Where outcomes were measured using different scales, the data were pooled and the effect measures were calculated using the standardized mean difference (SMD). For pooled dichotomous data, risk ratios (RR) with 95% CIs were calculated [27].

Meta-analyses results are presented in forest plots. If data are missing or are converted from statistics supplied, this is described in the footnotes. Data from each arm in a three-armed study [32] were used in the main comparison by halving the comparison group. Adverse effects were pooled into gastrointestinal disorders, generalized (systemic) adverse effects, all injection site disorders, and biochemical outcomes. Where quantitative synthesis of the data was not undertaken due to a lack of comparable intervention studies, or minimal reporting of outcomes in other studies, data are reported in narrative form.

Statistical heterogeneity between the studies was examined and reported using l^2^ and Chi^2^ statistics, with heterogeneity considered substantial if I^2^ > 50%. If ten or more studies reported data on the same outcome, publication or reporting bias was investigated by visual inspection of funnel plots for asymmetry.

### Subgroup and sensitivity analyses

Methodologic and clinical heterogeneity was explored using pre-specified meta-analysis, sensitivity, and subgroup analysis. A sensitivity analysis of trial design was undertaken, excluding trials that were at high risk for selection, performance, and detection bias. Sensitivity analysis also compared the effects of fixed-effects against random-effects modeling using the primary outcome fatigue, and the key secondary outcome hemoglobin concentration. Subgroup analyses were undertaken to look at the impact of baseline hemoglobin concentration (Hb ≤ 8.0 g/dL, 8.1–9.0 g/dL, 9.1–10.0 g/dL, ≥ 10.1 g/dL), and low (< 1000 mg) vs. high (≥ 1000 mg) doses of IV-iron on the outcomes of fatigue, hemoglobin, and ferritin parameters.

## Results

### Study selection

**Fig. 1:**
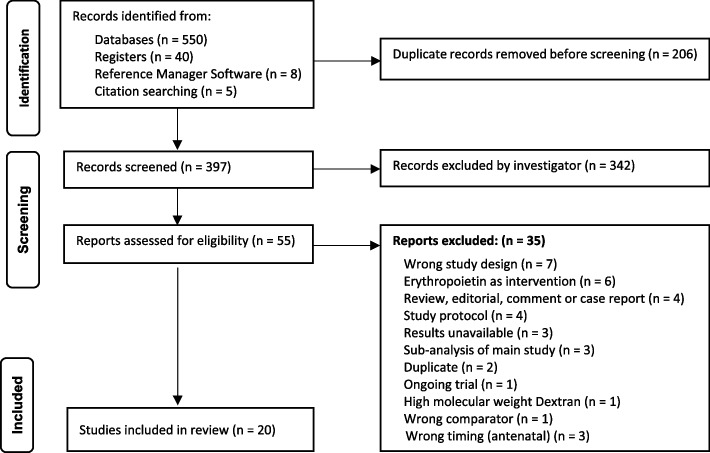
PRISMA flowchart including searches of databases, registers, and other methods

After the removal of duplicates, 397 studies were screened and 55 studies were assessed for eligibility, including 9 publications that were identified by hand-searches. The PRISMA diagram (Fig. 1) outlines screening, including reasons for exclusion. Twenty studies met the inclusion criteria for PPA interventions, all were randomized controlled trials:

- Fifteen studies compared IV-iron vs. oral iron for PPA (3,410 women) [32–46]
- One study compared IV-iron and oral iron vs. placebo and oral iron for PPA (60 women) [47]
- One study compared IV-iron and oral iron vs. oral iron for PPA (128 women) [48]
- One study compared IV-iron vs. RBC-T for PPA (13 women) [49]
- One study compared RBC-T vs. non-intervention for PPA (519 women) [12]
- One study compared single-unit vs. multiple-unit RBC-T for PPA (66 women) [50]

Our review comprised a total of 4196 women: 1834 allocated to IV-iron, 1771 allocated to oral iron, 330 allocated to RBC-T, and 261 allocated to non-intervention.

### Study characteristics

Characteristics of the included studies are displayed in Table 1. Postpartum hemoglobin concentration was the primary inclusion criterion for 15 of the studies [32–37, 39, 40, 42–48], with the upper threshold for inclusion ranging from Hb < 8 g/dL to < 11 g/dL. These levels included < 8 g/dL [39, 45, 47]; < 8.5 g/dL [48]; < 9 g/dL [33, 40]; < 10 g/dL [32, 35, 37, 42, 44]; < 10.5 g/dL [34], and < 11 g/dL [36, 43, 46]. Other primary inclusion criteria were postpartum hemorrhage [12, 38, 49]; requirement for blood transfusion [50], and (undefined) postpartum anemia [41].

**Table 1:** Characteristics of randomized controlled trials comparing the effectiveness and safety of interventions for postpartum anaemia

First author, year, country	Main eligibility criteria	Age (years) mean (SD)	Intervention n Control n Baseline Hb (g/dL),	Intervention: dosing and duration of exposure	Control: dosing and duration of exposure	Period of patient recruitment, and duration of follow-up	Primary Outcome
Bhandal 2006 United Kingdom [33]	Hb < 9 g/dL ferritin < 15 mcg/L	IV Iron: 29 (3.7) Oral iron: 28 (4.1)	IV Iron: *n* = 22 7.5 (0.8) Oral iron: *n* = 21 7.3 (0.9)	IV Ferric Sucrose, 200 mg D2 and D4 Total dose 400 mg	Oral ferrous sulphate 200 mg bd for 6 wks Mean total dose NR	24–48 h postpartum 6 wks	Hb, Hct, ferritin and serum iron on D5, D14 and D40
Breymann 2008 Switzerland, Russia, & Romania [34]	Hb < 105 g/L	IV Iron: 27.7 (5.5) Oral iron: 27.5 (5.4)	IV Iron: *n* = 227 9.7 (1.5) Oral iron: *n* = 117 9.7 (1.5)	IV FCM, up to 3 weekly doses (maximum 1000 mg per dose) Mean total dose 1346.7 g	Oral ferrous sulphate 100 mg bd for 12 wks Mean total dose NR	Up to 7 days postpartum 12 wks	Hb change at 12 wk
Damineni 2016 India [35]	Hb 7–10 g/dL	IV Iron: 28.0 Oral iron: 27.4	IV Iron: *n* = 45 8.7 (0.8) Oral iron: *n* = 45 8.9 (0.6)	IV FCM, single 1000 mg dose	Oral ferrous ascorbate 100 mg bd for 6 wks Mean total dose NR	D1 postpartum 6 wks	% to achieve a Hb ≥ 3 g/dL at 6 wks
Froessler 2013 Australia [36]	Hb < 11 g/dL and ferritin < 12 mcg/L	IV Iron: 28 (26–32) Oral iron: 30 (26–34)	IV Iron: *n* = 31 9.6^a^ (8.7–10.2) g/dL Oral iron: *n* = 43 9.5^a^ (8.9–10.6) g/dL	IV Ferric Sucrose, two 200 mg doses over 2 days at least 24 h apart Total dose 400 mg Folic acid 600 mcg daily for 6 weeks	Oral Ferrous sulphate 250 mg with 600 mcg folic acid bd for 6 wks Mean total dose NR	Within 72 h postpartum 6 wks	Hb and ferritin on D14 and 42
Guerra Merino 2011 Spain [37]	Hb 7–10 g/dL and ferritin < 15mcg/L	IV Iron: 34 Oral iron: 30	IV Iron: *n* = 6 8.9 (0.9) g/dL Oral iron: *n *= 7 8.6 (1.0) g/dL	IV Ferric Sucrose 200 mg D2 and D4 Total dose 400 mg	Oral ferrous sulphate 200 mg bd for 6 wks Mean total dose NR	24–48 h postpartum 6 wks	Hb, Hct, ferritin and serum iron on D7, D14 and D42
Hamm 2020 America [50]	Requires RBC-T, Hb < 7 g/dL or > 7 g/dL + signs or symptoms of anaemia	Single-unit RBC-T: 29 (6) Multiple-unit RBC-T: 29 (6)	Single-unit: *n* = 33 6.8 (0.6) g/dL Multiple Unit: n = 33 7.0 (0.6) g/dL	Single-Unit RBC-T Number of units of RBC-T: 1.2 (0.5)	Multiple-Unit RBC-T Number of units of RBCs: 2.1 (0.4)	Up to 4 days postpartum 4–9 wks postpartum	Total units of RBC-T
Holm 2017a Denmark [38]	PPH > 1000 mL and Hb 5.6—8.1 g/dL	IV-Iron: 30.4 (2.6) RBC-T: 34.5 (3.5)	IV-Iron: *n* = 7 6.80 (6.4–7.6) g/dL RBC-T: *n* = 6 6.80 (6.4–7.6) g/dL	IV-iron Isomaltoside Single 1500 mg dose Total dose 1500 mg	RBC-T units calculated from baseline Hb Five received 1-unit RBC-T, one received 2-units	≥ 12 h postpartum 12 wks	Change in physical fatigue score within 12 weeks
Holm 2017b Denmark [49]	PPH ≥ 700 mL and ≤ 1000 mL or PPH > 1000 mL and Hb > 6.5 g/dL	IV-Iron: 32.2 (4.4) Oral iron: 32.6 (4.5)	IV-Iron: *n* = 97 9.71 g/dL Oral iron: *n* = 90 9.71 g/dL	IV-iron Isomaltoside Single 1200mg dose	Variable oral iron dosing and timing. Formulation not stated Mean total dose 4784 mg (4309)	≥ 12 h postpartum 12 wks	Change in physical fatigue score within 12 weeks
Jain 2013 India [39]	Hb < 8 g/dL within 48 h postpartum	IV Iron: 24 (3.5) Oral iron: 25 (2.7)	IV Iron: *n* = 21 6.7 (0.3) Oral iron: *n* = 20 6.8 (0.2)	IV iron sucrose 100–200 mg three times in one week Total dose 300–600 mg	Oral ferrous fumarate 300 mg for 2 wks Mean total dose NR	24–48 h postpartum 2 wks	Change in Hb on D7 and D14
Mumtaz 2011 India [40]	Hb < 9 g/dL, ferritin < 15 mcg/L	IV Iron: 24.6 (3.7) Oral iron: 25.1 (3.3)	IV Iron: *n* = 40 8.4 (0.3) Oral iron: *n* = 40 8.1 (0.5)	IV-iron sucrose Two 200 mg doses on D2 and D4 Total dose 400 mg	Oral ferrous sulphate 200 mg bd for 6 wks Mean total dose NR	24–48 h postpartum 40 days	Hb, Hct, red-cell indices, ferritin and serum iron on D7, D14 and D40
Perello 2014 Spain [47]	Hb 6.0–8.0 g/dL	IV and oral iron: 29.5 (5.8) Oral iron and placebo: 29.9 (5.3)	IV and oral iron: *n* = 31 7.2 (0.5) g/dL Oral iron and placebo: *n* = 29 7.3 (0.5) g/dL	IV-iron sucrose & oral ferrous sulphate 525 mg Two × 200 mg of IV-iron on consecutive days, & bd oral iron for 30 days Mean total dose NR	Placebo and oral ferrous sulphate 525 mg Placebo and bd oral iron for 30 days Mean total dose NR	≤ 48 h postpartum 6 wks	Change in Hb and Hct at 6 wks
Prick 2014 Netherlands [12]	PPH ≥ 1000 mL and/or Hb drop ≥ 1.9 g/d/L, *and* Hb 4.8–7.9 g/dL	RBC-T: 30.7 (5.0) Non-intervention: 30.9 (5.3)	RBC-T: *n* = 258 7.3 (6.8–7.7) Non-intervention: *n* = 261 7.4 (6.8–7.7)	RBC-T At least one unit of RBC-T	Non-intervention (n = 261) Oral and IV iron according to local protocol. RBC-T if severe symptoms	12–24 h postpartum 6 wks	Physical fatigue at D3 postpartum
Rathod 2015 India [32]	Hb < 10 g/dL	IV-iron FCM: 25.9 (3.6) IV-iron sucrose: 26.0 (3.7) Oral iron: 25.4 (3.1)	IV-iron FCM: *n* = 100, 7.71 (1.17) IV-iron sucrose: *n* = 100, 8.05 (1.07) Oral iron: *n* = 100 8.23 (1.01)	IV-iron FCM Calculated dose not more than once a week Mean total dose NR IV-iron sucrose Calculated dose on alternate days Mean total dose NR	Oral ferrous ascorbate 100 mg od Duration of exposure unclear Mean total dose NR	﻿Recruitment period unclear 6 wks	Changes in Hb and ferritin levels at 2 wks and 6 wks
Razzaq 2017 Pakistan [41]	Postpartum anaemia (not defined)	IV-Iron: 26.36 (4.3) Oral iron: 26.31 (4.7)	IV-Iron: *n* = 41 Stratified by baseline^c^ Hb ≤ 7: 53% Hb 71–99: 47% Oral iron: *n* = 41 Hb ≤ 7: 44% Hb 71–99: 56%	IV-iron formulation unclear Up to 1000 mg given, repeated weekly Mean total dose NR	Oral Ferrous sulphate 325 mg tds for 6 wks Mean total dose NR	Recruitment period unclear for 6 wks	% to achieve Hb rise > 3.5 g/dL at 6 wks
Seid 2008 USA [42]	Hb < 10.0 g/dL within 10 days of birth	IV-Iron: 26.39 (5.97) Oral iron: 26.49 (5.55)	IV-iron: *n* = 139 8.91 (0.89) g/dL Oral iron group: *n* = 147 8.88 (0.89) g/dL	IV-iron FCM Given up to 2500 mg in divided weekly doses (maximum 1000 mg per dose) Mean dose 1503.5 mg	Oral Ferrous sulphate 325 mg tds for 6 wks Mean dose 7906.1 mg	≤ 10 days postpartum for 6 wks	% to achieve Hb > 12 g/dL
Seid 2017^b ^USA [43]	Hb ≤ 11.0 g/dL	IV-Iron: 31.2 (9.36) Oral iron: 31.4 (8.98)	IV-iron: *n* = 606 10.2 (1.16) g/dL Standard Medical Care: *n* = 623 10.11 (1.19) g/dL	IV-iron FCM Single dose up to 1000mg Mean dose 926 mg	Standard medical care for 30 days using oral ferrous sulphate 325 mg tablets if given Mean total dose NR	≥ 18 h postpartum 30 days	﻿Incidence of serious AEs, including death, hospitalization, disability, congenital anomaly/birth defect, and life-threatening events
Vanobberghen 2021 Tanzania [46]	Hb < 11.0 g/dL and ferritin < 50 mcg/L	IV-Iron: 26^a^(22–30) Oral iron: 26 (22–31)	IV Iron: *n* = 114 9.8 (8.6–10.5) g/dL Oral iron: *n* = 116 9.2 (8.5–10.3) g/dL	IV-iron FCM Total dose calculated Mean total dose NR	Oral ferrous sulphate 200 mg tablets & 5 mg folic acid tds until 3 months after correction of anaemia Mean total dose NR	≤ 14 days postpartum 12 months	% to achieve a Hb > 11.5 g/dL at 6 weeks
Van Wyck 2007 USA & Mexico [44]	Hb ≤ 10 g/dL	IV-Iron: 26.9 (6.4) Oral iron: 26.1 (6.0)	IV Iron: *n* = 174 9.0 (0.9) g/dL Oral iron: *n* = 178 9.0 (1.0) g/dL	IV FCM Total dose up to 2500 mg, maximum 1000 mg per dose given weekly Mean dose 1403.1 mg	Oral ferrous sulphate 325 mg tds for 6 wks Mean dose 6764 mg	≤ 10 days postpartum 6 wks	% to achieve a Hb rise ≥ 2 g/dL
Verma 2011 India [45]	Hb < 8 g/dL	IV-Iron: 25 (2.0) Oral iron: 24 (3.0)	IV-iron: *n* = 75 7.58 g/dL Oral iron: *n* = 75 7.42 g/dL	IV-iron sucrose 600–800 mg in three 200 mg doses on alternate days Mean total dose NR	Oral ferrous sulphate 200mg bd for 4 wks Mean total dose NR	≤ 24 h postpartum 4 wks	Change in Hb
Westad 2008 Norway [48]	Hb ≥ 6.5 to ≤ 8.5	IV-Iron: 29.5 (4.1) Oral iron: 29.6 (4.5)	IV and oral iron: *n *= 58 7.91 (6.9) g/dL Oral iron: *n* = 70 7.72 (8.4) g/dL	IV-iron sucrose 600 mg in three 200 mg doses After 4 wks, bd oral ferrous sulphate 100 mg Mean total dose NR	Oral ferrous sulphate 100 mg bd for 12 wks Mean total dose NR	≤ 48 h postpartum 12 wks	Change in Hb at 4 wks

*Bd* twice daily, *D* Postpartum day, *FCM* Ferric carboxymaltose, *Hb* Hemoglobin concentration, *Hct* Haematocrit, *IV* Intravenous, *mo* Months, NR Not reported, *od* Once daily, *po* oral, *RBC-T* Red blood cell transfusion, *SD* Standard deviation, in brackets, *tds* 3 times daily, *RCT* Randomised controlled trial, *wks* Weeks

^a^median (inter-quartile range)

^b^ includes women with heavy menstrual bleeding

^c^No mean baseline

Baseline hemoglobin concentrations varied significantly across studies. Nineteen studies reported a pre-intervention baseline mean or range. These studies included: Hb ≤ 7.0 g/dL [39, 49, 50]; 7.1 − 8.0 g/dL [12, 32, 33, 45, 47, 48]; 8.1–9.0 g/dL [35, 37, 40, 42, 44]; 9.1 − 10.0 g/dL [34, 36, 38, 46, 51], and ≥ 10.1 g/dL [43]. The remaining study stratified participants by baseline hemoglobin concentration, with no mean hemoglobin concentration reported [41].

### Intravenous iron preparations and dosing regimens

Of the eighteen studies with IV-iron intervention arms, IV ferric sucrose was the formulation in nine studies [32, 33, 36, 37, 39, 40, 45, 47, 48]; IV ferric carboxymaltose in seven studies [32, 34, 35, 42–44, 46], and IV-iron isomaltoside in 2 studies [38, 49]. The IV-iron formulation was not stated in one study [41]. Nine studies with IV-iron intervention arms had fixed doses of IV-iron: five studies used 400 mg IV-iron sucrose [33, 36, 37, 40, 47]; one of 600 mg IV-iron sucrose [48]; one of 1000 mg of [35]; one of 1200 mg of IV-iron Isomaltoside [38], and one of 1500 mg of IV-iron Isomaltoside [49]. The dose of IV iron sucrose was unclear in one study [45]. The dose of up to 1000 mg of ferric carboxymaltose was adjusted for body weight in one study [43], and for body weight and hemoglobin concentration in one study [46].

Five studies with IV-iron intervention arms specified target hemoglobin (Hb) concentration for the treatment of PPA: one study had a target Hb of 12 g/dL [32]; two studies had a target Hb of 15 g/dL [42, 44], and one study had a target of Hb 12 − 16 g/dL [34]. One study calculated the IV-iron dose on an unspecified target hemoglobin concentration [39]. In one study, up to 1000 mg of IV-iron was given weekly, the maximum dose or method of calculation was not stated [41].

### Oral iron preparations and dosing regimens

Of the seventeen studies with oral iron intervention arms, ferrous sulfate was the formulation in thirteen studies [33, 34, 36, 37, 40–48]; ferrous ascorbate in two studies [32, 35]; ferrous fumarate in one study [39], and an unstated formulation in one study [38]. The duration of exposure to oral iron ranged from 14 days [39] to 12 weeks [38]. For one study with a 12-month follow-up, oral iron was taken for 3 months after correction of anemia [46]. The per protocol elemental iron regimens ranged from 1400 mg [39] to 8400 mg [35]. Adherence or compliance to oral iron was reported as 100% [33, 39], ≥ 95% [42, 43], ≥ 90% [34, 46], 84% [35, 44], 51% [32], < 50% [48], good [37], not good [45], satisfactory [40], or not stated [36, 38, 41]. The mean dose of oral iron was stated in only three studies [38, 42, 44] (Table 1).

### Red blood cell transfusion

Three studies had RBC-T intervention arms [12, 49, 50]. In one study [49] the number of units of RBC-T was determined by baseline hemoglobin concentration: women with Hb 5.6–6.3 g/dL received 2 units, and women with Hb 6.4–8.1 g/dL received 1 unit. One study [50] randomized eligible women to single or multiple units of RBC-T. One study [12] allocated at least one unit in the RBC-T intervention arm. Of the 17 studies with IV-iron and oral iron intervention arms, 11 had peripartum RBC-T as exclusion criteria [32, 33, 35, 37, 39–44, 47]; the requirement for RBC-T was an outcome in three studies [34, 36, 38]. Results from one study [48] included 6.9% and 14.3% of women who received RBC-Ts in the IV-iron and oral iron intervention arms respectively.

### Risk of Bias

**Fig. 2:**
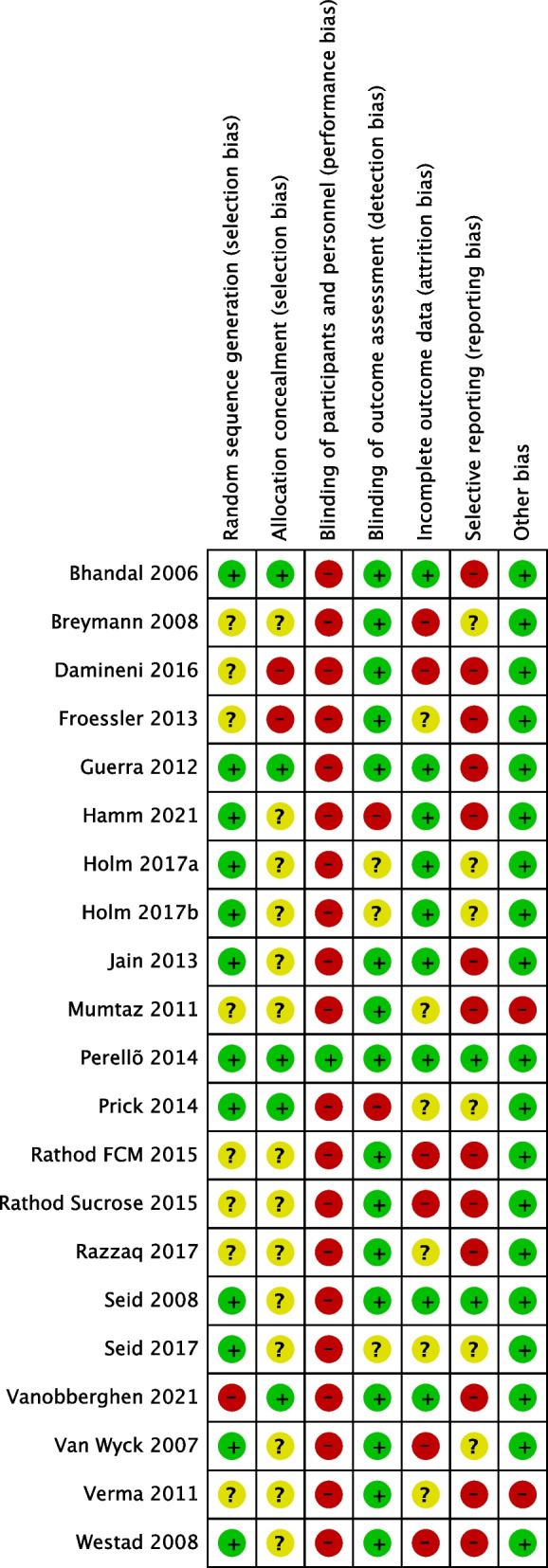
Risk of bias assessment for included studies

Nineteen studies were unblinded and therefore at high risk of bias. One study [47] was blinded and had a low risk of bias across all domains. The risk of bias assessment for included studies is summarised in Fig. 2.

### Synthesis of results

Fifteen studies of IV-iron vs. oral iron [32–46] were available for meta-analysis.

### Primary outcome: fatigue

**Fig. 3:**
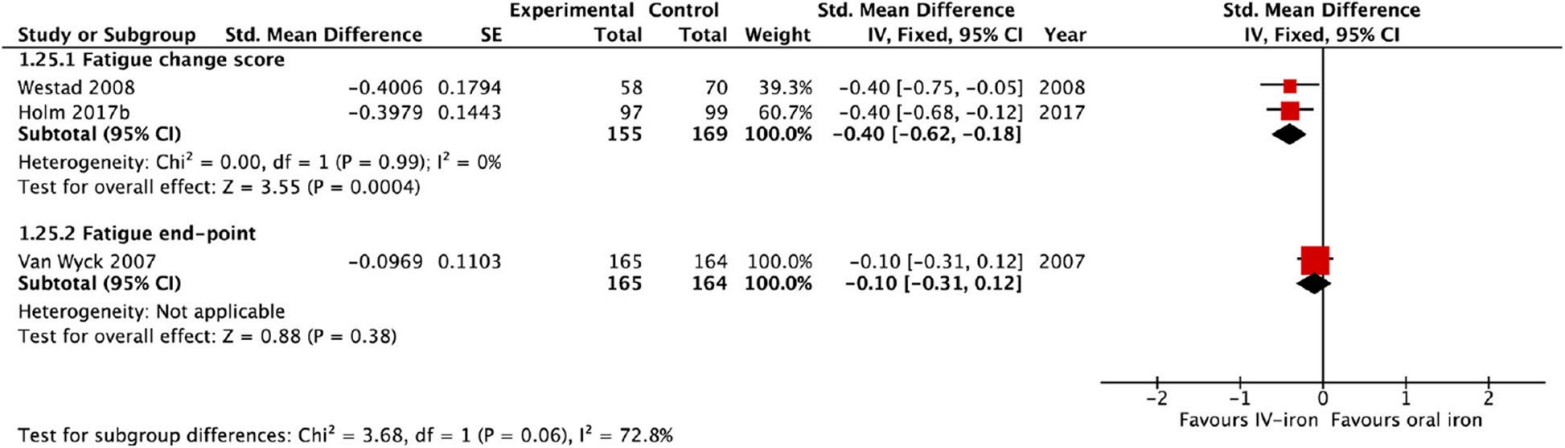
Forest plot for comparison of IV-iron vs. oral iron: fatigue

Six studies (with 1251 participants) on interventions for PPA [12, 38, 44, 48–50] reported fatigue as an outcome: three of IV-iron vs. oral iron [38, 44, 48]; one of RBC-T vs. non-intervention [12]; one of IV-iron vs. RBC-T [49]; and one of single-unit vs. multiple-unit RBC-T for PPA [50]. The three studies of IV-iron vs. oral iron used different fatigue scales. Two reported changes in scores, using either The Fatigue Scale [52] or the Multidimensional Fatigue Inventory (MFI) [53]. In the meta-analysis, there was a significant reduction in fatigue with IV-iron, compared to oral iron (SMD − 0.40, 95% confidence interval (CI) − 0.62, − 0.18, *I*^2^ = 0%) (Fig. 3), with a low certainty of the evidence (Table 3). One study [44] reported visual end-point data using the Fatigue Linear Analogue Scale. There were no significant differences in fatigue scores between the IV-iron and oral-iron groups at 14 or 42 days (Fig. 3).

The study of RBC-T vs. non-intervention for PPA [12] reported physical fatigue with the MFI. After adjusting for baseline fatigue and mode of birth, women with non-intervention had significantly higher mean fatigue scores at 1 week (MD 1.06, 95% CI 0.3, 1.8, *p* = 0.01), although non-inferiority was not demonstrated by the predetermined difference of 1.3 [12]. A pilot trial of IV-iron vs. RBC-T [49] reported no difference in the primary outcome of physical fatigue score at 12 weeks using the MFI (mean difference (MD) − 0.63, 95% CI − 3.28, 2.02, *p* = 0.61), and a trial of single vs. multiple units of RBC-T also reported no difference in median general fatigue scores at 4–9 weeks (*p* = 0.13) using the MFI [50].

### Hemoglobin parameters

**Fig. 4:**
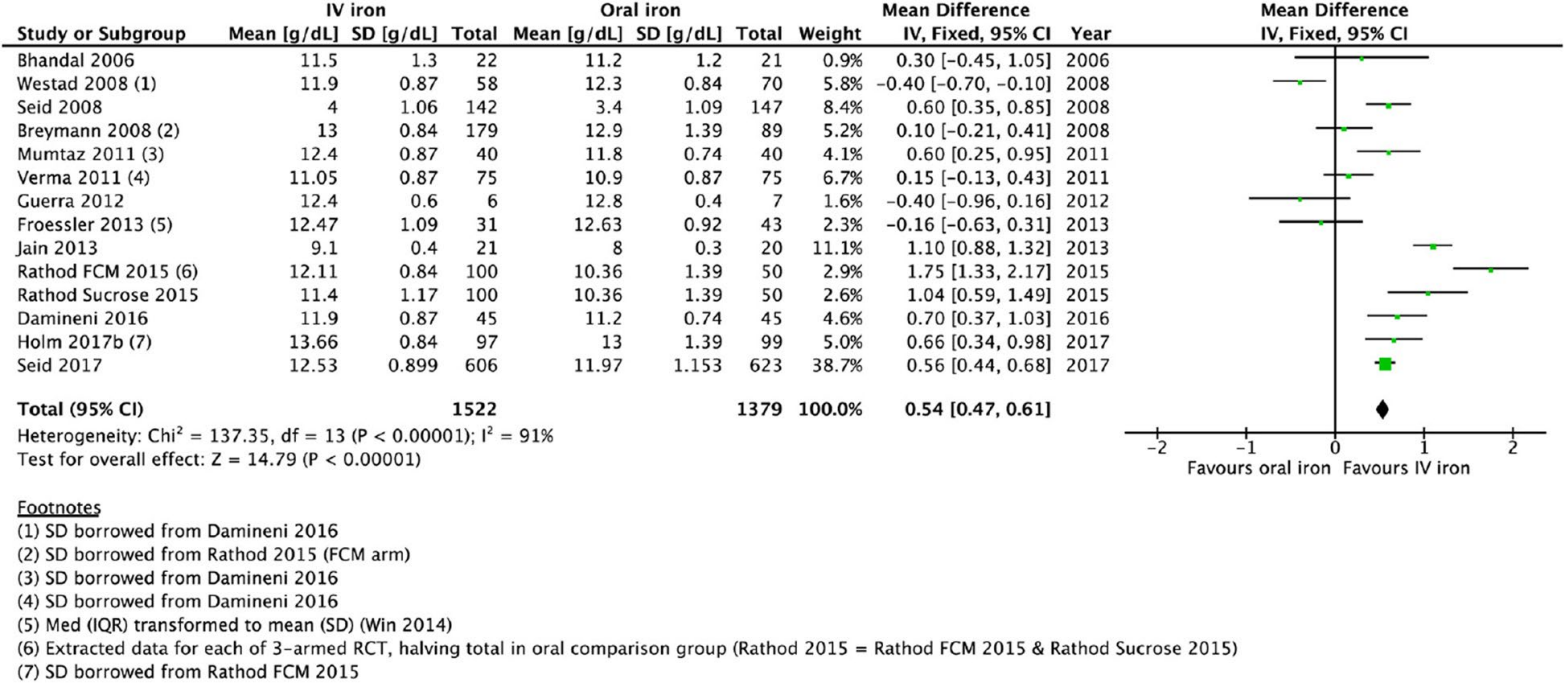
Forest plot for comparison of IV-iron vs. oral iron: Hb concentration longest follow-up (g/dL)

Meta-analysis was undertaken on thirteen studies comparing IV-iron against oral iron for PPA [32–40, 42, 43, 45, 48]. In addition, Westad et al. [48] commenced oral iron in the IV-iron intervention arm after 4 weeks, therefore Hb concentration data at 4 weeks were available for this meta-analysis. The MD in Hb concentration was significantly higher in the IV-iron group at the longest follow-up (0.54 g/dL, 95% confidence interval (CI) 0.47, 0.61, *I*^2^ = 91%) (Fig. 4) with a moderate certainty of the evidence (Table 2).

**Table 2.**
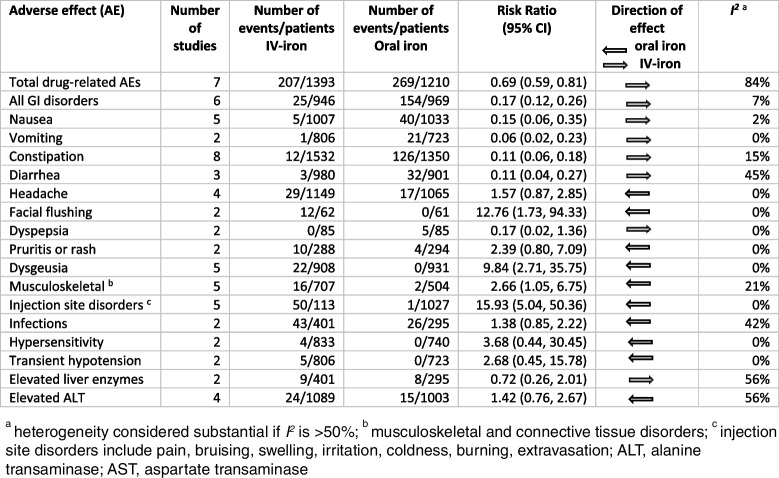
Drug-related adverse effects for comparison of intravenous and oral iron for postpartum anemia

Sensitivity analysis with random effect modeling found a similar effect on Hb concentration at the longest follow-up, favoring IV-iron (MD 0.48 g/dL, 95% CI 0.23, 0.74, *I*^2^ = 91%). In a post-hoc sensitivity analysis of Hb concentration at the longest follow-up, excluding studies [34, 36, 38, 43, 48] with RBC-T use in the IV-iron and/or oral intervention arms, IV-iron was significantly more effective at increasing Hb concentration than oral iron, although heterogeneity remained high (MD 0.73 g/dL, 95% CI 0.62, 0.84,* I*^2^ = 89%) (Additional Figure S1). Subgroup analysis of low and high IV-iron dosing regimens found no difference (*p* = 0.82,* I*^2^ = 0%) in Hb concentration at the longest follow-up between subgroups (Additional Figure S2).

Subgroup analysis of different mean Hb baseline concentrations found the difference in effect between baseline Hb subgroups was significant (*p* = 0.009;* I*^2^ = 74%). IV iron had more of an effect in the subgroup with a baseline Hb concentration of 8.1–9.0 g/dL than those with lower *and* higher baseline Hb concentrations (MD 0.60 g/dL, CI 0.44, 0.75; Additional Figure S3). In a further sensitivity analysis of baseline subgroups that excluded two studies [36, 48] with RBC-T use, the difference between baseline Hb concentration subgroups was greater (*p* < 0.0001, *I*^2^ = 86%); the most significant improvement in Hb concentration after IV-iron was in the lowest baseline group of Hb ≤ 8 g/dL (MD 0.86 g/dL, CI 0.71, 1.02) compared to all other baseline groups (Additional Figure S4).

Meta-analysis of four studies [35, 41, 42, 44] assessing the proportion of women achieving an increase in Hb ≥ 2 g/dL from baseline favored IV-iron over oral iron (risk ratio (RR) 1.22, 95% CI 1.15, 1.31, *I*^2^ = 95%). Meta-analysis of four studies [32, 34, 42, 44] assessing the proportion achieving a rise in Hb ≥ 12.0 g/dL favored IV-iron over oral iron (RR 1.37, 95% CI 1.27, 1.48, *I*^2^ = 89%).

For the studies undergoing meta-analyses, heterogeneity was very high for all hematological outcomes (*I*^2^ = 74–99%). We did not observe evidence of publication bias in the funnel plot of studies included in a meta-analysis of Hb concentration (Additional Figure S5).

Four studies reporting Hb concentration could not be included in the meta-analysis due to different comparison groupings. The pilot study of IV-iron vs. RBC-T [49] agreed with the overall finding that IV-iron was associated with significantly higher Hb concentration at the longest follow-up (*p* < 0.05). The blinded study of IV-iron and oral iron vs. placebo and oral iron [47] found no significant difference in Hb concentration at 6 weeks (MD − 0.03, 95% CI − 0.6, 0.6). The study of RBC-T vs. non-intervention [12] found no significant difference in Hb concentration at 6 weeks (*p* < 0.18) although additional oral and IV-iron was permitted, with a higher percentage of participants in the non-intervention group than the RBC-T group receiving oral (76% vs. 40% participants) and IV-iron (12% vs. 0% participants) [12]. The study of single-unit vs. multiple-unit RBC-T [50] found significantly higher Hb concentration prior to hospital discharge in the multiple-unit RBC-T group (MD − 0.7, 95% CI 1.06, − 0.34). The single-unit RBC-T arm used significantly more IV-iron (46%) than the multiple-unit RBC-T arm (21%) (RR 2.14, 95% CI 1.01, 4.57); hemoglobin concentration was not reported beyond discharge [50].

### Ferritin concentration

**Fig. 5:**
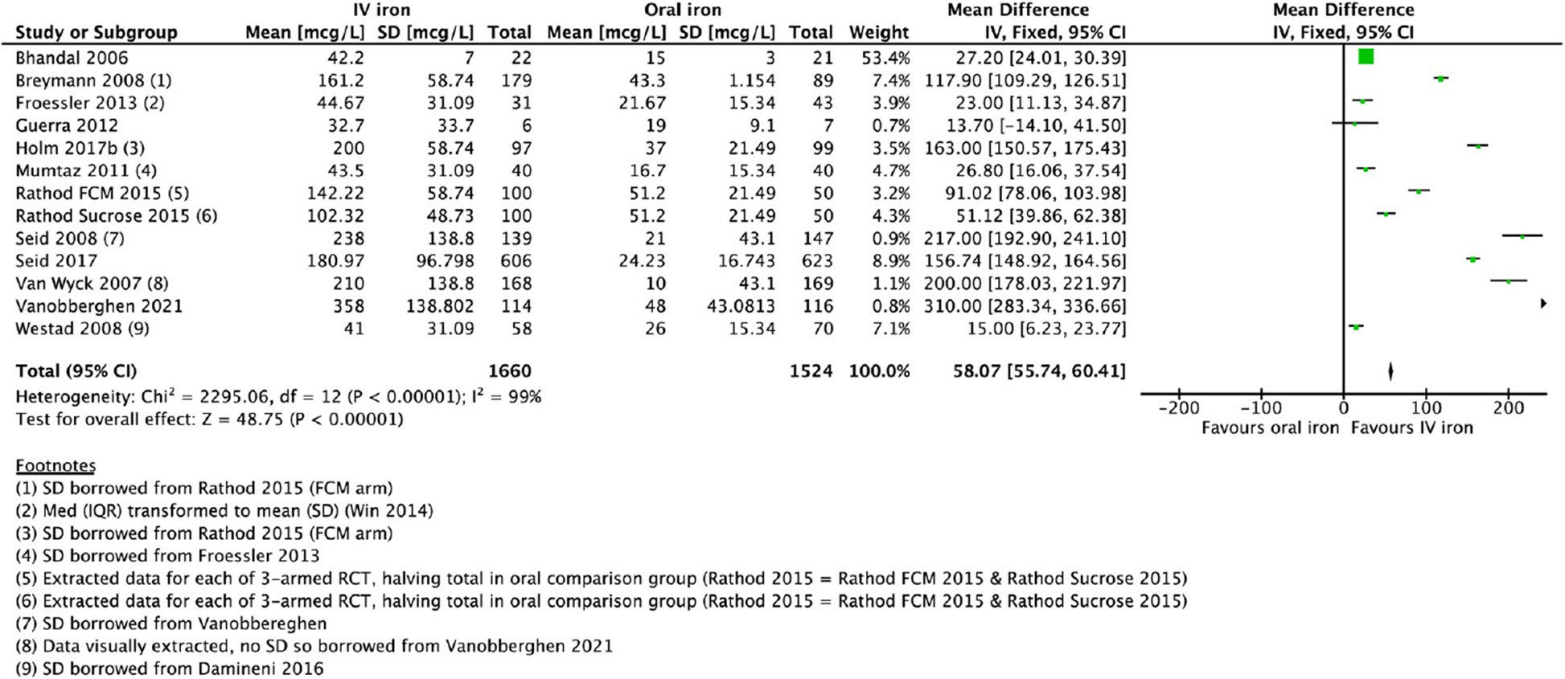
Forest plot for comparison of IV-iron vs. oral iron: ferritin concentration longest follow-up (mcg/L)

Meta-analysis was undertaken on twelve studies of IV-iron vs. oral iron for PPA that reported ferritin concentration [32–34, 36–38, 40, 42–44, 46, 48]: the direction of the effect favored IV-iron (MD 58.07 mcg/L, 95% CI 55.74, 60.41, *I*^2^ = 99%) with a moderate certainty of the evidence (Fig. 5). Two other studies reporting ferritin concentration could not be included in the meta-analysis as they were different treatment comparisons. The pilot study of IV-iron vs. RBC-T [49] agreed with the overall finding that IV-iron was associated with higher ferritin concentration at the longest follow-up: this was significant at 7 days (*p* < 0.05), and the mean ferritin was 141 mcg/L at 12 weeks although comparative data were not available [49]. Ferritin concentration in the RBC-T group remained low throughout the study and was below normal at 12 weeks. The study of IV-iron and oral iron vs. placebo and oral iron [47] found no significant difference in ferritin concentration at 6 weeks (MD 17.2, 95% CI − 8.4, 42.8). Two other studies [12, 50] with RBC-T as an intervention did not report ferritin concentration as an outcome.

### Adverse effects and symptoms

Meta-analysis was undertaken on seven studies [32, 34, 37, 38, 42, 43, 46] reporting total drug-related adverse events in the comparison of IV-iron and oral iron for PPA. Overall, the risk ratio was significantly lower for IV-iron (0.69, 95% CI 0.59, 0.81, *I*^*2*^ = 84%) (Additional Figure S6). Specific drug-related adverse events in the comparison of IV-iron and oral iron for PPA are outlined in Table 2 and presented in forest plots in Additional Figures S7–S12. In the oral-iron group, there were statistically significantly higher frequencies of nausea, vomiting, constipation, and diarrhea, compared to the IV-iron group. In the IV-iron group, there were statistically significant higher frequencies of facial flushing, dysgeusia (altered taste), musculoskeletal disorders (e.g., myalgia), hypophosphatemia, and injection site disorders, compared to the oral iron group. There were no statistically significant differences in frequencies of headaches, dyspepsia, pruritis, infections, hypersensitivity, transient hypotension, or elevated liver enzymes between the IV-iron and oral iron groups (Additional Figures S7–S12). Four cases of hypersensitivity to IV-iron were reported in two studies comparing IV-iron and oral iron for PPA [34, 43] (Table 2). No cases of anaphylaxis were reported. One woman died of non-drug-related peripartum cardiomyopathy 7 days after receiving ferric carboxymaltose [44].

Table 3 summarizes the pooled analyses of findings and the quality of the evidence of drug-related adverse events when comparing IV-iron with oral iron for PPA. Heterogeneity was low for all gastrointestinal (*I*^2^ = 7%) and injection site (*I*^2^ = 0%) adverse events.

**Table 3:** Summary of findings for IV-iron compared to oral iron for postpartum anemia

Outcomes	Anticipated absolute effects^* ^(95% CI)		Relative effect (95% CI)	№ of participants (studies)	Certainty of the evidence (GRADE)	Comments
	Risk with oral iron	Risk with IV Iron				
Fatigue change score	–	SMD **0.4 lower** (0.62 lower to 0.18 lower)	–	324 (2 RCTs)	⊕⊕OO Low^a,b^	IV Iron may result in a slight reduction in Fatigue change score
Fatigue end-point	–	SMD **0.1 lower** (0.31 lower to 0.12 higher)	–	329 (1 RCT)	⨁⨁OO Low^c,d^	IV Iron may result in little to no difference in Fatigue end-point
Hemoglobin concentrations (change and endpoint scores): longest follow-up	The mean Hb levels (change and endpoint scores): longest follow-up was **11.5** g/dL	MD **0.54 g/dL higher** (0.47 higher to 0.61 higher)	–	2901 (13 RCTs)	⊕⊕⊕O Moderate^e^	IV Iron likely results in an increase in Hb concentrations (change and endpoint scores): longest follow-up
Ferritin concentration (change and endpoint scores): longest follow-up	The mean ferritin concentration (change and endpoint scores): longest follow-up was **29.6** mcg/L	MD **58.07 mcg/L higher** (55.74 higher to 60.41 higher)	–	3184 (12 RCTs)	⊕⊕O Moderate^f^	IV Iron likely results in an increase in ferritin concentration (change and endpoint scores): longest follow-up
Total drug-related adverse effects	222 per 1000	**153 per 1000** (129 to 180)	**RR 0.69** (0.58 to 0.81)	2603 (7 RCTs)	⊕⊕OO Low^g,h^	The evidence suggests that IV iron reduces total drug-related AEs
All gastrointestinal disorders	159 per 1000	**27 per 1000** (19 to 41)	**RR 0.17** (0.12 to 0.26)	1915 (6 RCTs)	⊕⊕⊕⊕ High^i^	IV iron results in fewer GI disorders than oral iron
Headache	16 per 1000	**25 per 1000** (14 to 45)	**RR 1.58** (0.86 to 2.83)	2214 (4 RCTs)	⊕⊕⊕ OO Moderate^j^	IV iron probably results in an increase in headaches
Dysgeusia (taste distortion)	0 per 1000	**0 per 1000** (0 to 0)	**RR 9.84** (2.71 to 35.75)	1839 (5 RCTs)	⊕⊕⊕⊕ High^k^	IV iron results in a large increase in frequency of dysgeusia (taste distortion)
Musculoskeletal disorders, e.g., myalgia	4 per 1000	**11 per 1000** (4 to 27)	**RR 2.66** (1.05 to 6.75)	1211 (5 RCTs)	⊕⊕⊕ O Moderate^l^	IV iron likely results in an increase in musculoskeletal disorders
All injection site disorders-Injection site pain, bruising, swelling, irritation, burning, or reaction	1 per 1000	**16 per 1000** (5 to 49)	**RR 15.93 **(5.04 to 50.36)	2140 (5 RCTs)	⊕⊕⊕ O Moderate^l^	IV iron results in large increase in injection site disorders

*CI* confidence interval, *MD* mean difference, *RR* risk ratio, *SMD* standardized mean difference

GRADE Working Group grades of evidence

*High certainty*: we are very confident that the true effect lies close to that of the estimate of the effect

*Moderate certainty*: we are moderately confident in the effect estimate: the true effect is likely to be close to the estimate of the effect, but there is a possibility that it is substantially different

*Low certainty*: our confidence in the effect estimate is limited: the true effect may be substantially different from the estimate of the effect

*Very low certainty*: we have very little confidence in the effect estimate: the true effect is likely to be substantially different from the estimate of effect

*Explanations*

^a^Downgraded one level because both studies unblinded, and unclear allocation concealment

^b^Downgraded one level for precision because there were < 400 participants in the fatigue outcome, and both studies had varying off-protocol RBC-T use in each study arm

^c^Downgraded one level because unblinded, and unclear allocation concealment

^d^Downgraded one level for precision because there were < 400 participants in the fatigue outcome

^e^Downgraded one level for inconsistency as *I*^2 ^= 91% and different studies have different conclusions

^f^Downgraded one level for inconsistency as *I*^2 ^= 99%

^g^We downgraded one level because all 7 studies unblinded; it was unclear if allocation was concealed in 5/7 studies

^h^Downgraded one level for inconsistency as *I*^2 ^= 86% and different studies have different conclusions

^i^We downgraded one level because all 6 studies unblinded; allocation concealment was unclear for 4/6 studies

^j^We downgraded one level because unblinded, and unclear allocation concealment in all 4 studies

^k^We downgraded one level because all 5 studies unblinded; it was unclear if allocation was concealed in 4/5 studies

^l^We downgraded one level because all 5 studies unblinded and unclear allocation concealment

Reported injection-site disorders included pain, bruising, swelling, irritation, coldness, burning, and extravasation. IV-iron site discoloration (skin staining) was reported separately as this has potential long-term consequences. Of the IV-iron and oral-iron comparison studies, one study [38] reported IV-iron site discoloration (RR 7.14, 95% CI 0.37, 136.47). IV-iron site discoloration was also reported in the study [49] of IV-iron vs. RBC-T (RR 2.63, 95% CI 0.13, 54.64). The pilot study [49] reported more total drug-related adverse effects in the IV-iron group compared to the RBC-T group (RR 1.29, 95% CI 0.31, 5.31); also reported a high (2/6, 33%) rate of transfusion-related pyrexia in the RBC-T group. In one study [47] there were more drug-related adverse effects in the placebo and oral iron group, compared to the IV and oral iron group (RR 0.37, 95% CI 0.08, 1.78).

The study comparing RBC-T with non-intervention [12] reported 1.3% (3/227) transfusion reactions in the RBC-T arm: one rash and two cases of pyrexia. There was no significant difference in infectious complications or thromboembolic events between groups (RR 0.02, 95% CI 0.00, 0.36) [12]. The study of single-unit vs. multiple-unit RBC-T for PPA [50] reported no transfusion reactions and no difference in frequencies of infection, endometritis, venous thromboembolism, intensive care admission, hospital readmissions, or length of hospital stay [50].

### Signs and symptoms of anemia

Perello et al. [47] reported more anemia symptoms in the IV and oral iron group compared to the placebo and oral iron group at 6 weeks (RR 2.81, 95% CI 0.31, 25.48). Prick et al. [12] reported more anemia symptoms in the non-intervention group compared to the RBC-T group (RR 56.35, 95% CI 3.46, 918.07). Hamm et al. found no significant differences in pre and post-intervention dizziness/fatigue (*p* = 1.00), heart rate (*p* < 0.08), systolic (*p* < 0.66), and diastolic blood pressures (*p* < 0.73) between single-unit vs. multiple-unit RBC-T interventions for PPA at 4–6 h post-transfusion [50].

### Breastfeeding

In comparison of IV-iron and oral iron, there was no difference in time to lactogenesis (*p* = 0.78) and time to discontinuation of breastfeeding (*p* = 0.52) [38]. There was a significant but transient difference in breast milk iron concentration between the IV-iron and oral iron groups (*p* < 0.001) that disappeared after 1 week (*p* = 0.64) [54]. No difference was found in breastfeeding rates between single-unit (61.5%) and multiple-unit (63.6%) RBC-T groups for PPA (*p* = 0.89) [50].

### Postnatal depression and quality of life outcomes

Across different comparison groups, four studies [38, 47, 49, 50] reported postnatal depression as an outcome following interventions for PPA. There was a significant improvement in Edinburgh Postnatal Depression Scores (EPDS) in favor of IV-iron compared to oral iron at 1, 3, and 8 weeks postpartum (*p* = 0.05) [38]. There was no difference in risk of depression (EPDS ≥ 11) between the IV and oral iron group, compared to the placebo and oral iron group (MD − 0.1, 95% CI − 0.3, 0.1) [47].

No difference in EPDS was found in comparison to IV-iron and RBC-T [49]. Between the single-unit and multiple-unit RBC-T groups, there were no differences in EPDS (*p* = 0.34) and the Maternal Attachment Inventory (*p* = 0.55) [50].

Two studies [44, 48] comparing IV-iron and oral iron for PPA reported quality of life outcomes using the SF-36 [55]. One study [44] found no significant differences between groups at any time-point. One study [48] found a significant difference in the SF-36 pain index at 12 weeks, favoring IV-iron (*p* = 0.03). A significant improvement in physical functioning was found in the RBC-T group compared to non-intervention at 1 week (MD − 5.5, 95% CI − 10.3, − 0.7, *p* < 0.05) and 6 weeks (MD − 4.3, 95% CI − 8.4, − 0.2, *p* < 0.05) postpartum, using a SF-36 sub-scale [12]. There were no significant differences between groups in other SF-36 dimensions [12].

## Discussion

This systematic review and meta-analysis examined data from 20 randomized trials of IV-iron and/or RBC-T for the treatment of PPA. The primary outcome was maternal fatigue, with hemoglobin and ferritin concentrations, and other quality-of-life measures as secondary outcomes. This differs from other systematic reviews of PPA as it focuses on a women-centered outcome, as well as hematological outcomes.

Our findings suggest women with PPA have less fatigue if treated with IV-iron compared to oral iron, or RBC-T compared to non-intervention. However, the overall quality of the evidence was very low due to limited reporting and the use of different fatigue scales limited the meta-analysis. Four [12, 38, 49, 50] of the six studies reporting fatigue used the MFI. Although this has been evaluated as a feasible and reliable tool [25], the minimum clinically important difference for fatigue using the MFI is yet to be determined [12, 38, 49] adding to the uncertainty of evidence. The measurement of fatigue appears to be challenging, likely due to the complexity of the phenomenon.

Physical fatigue is the earliest complaint from women with acute anemia [12]. Treatment of fatigue by correction of iron-deficiency anemia may be a biological pathway to prevent and reduce postpartum depression [18, 56]. However, only four studies from different treatment comparisons reported maternal depression and only one found an improvement in the EPDS with IV-iron compared to oral iron. Given the association between maternal anemia and depression [21–24, 57] and the impact of depression on women, infants, and families, more evidence on PPA interventions as a pathway to reduce the risk of postpartum depression is required.

Breastfeeding is an outcome that is of central importance to women, but the data are too limited to draw conclusions. PPA is likely to impact breastfeeding, although evidence is limited to one small study [58]. The ability to recover and breastfeed is sometimes used by clinicians as part of the decision-making for prescribing RBC-T [50]; however, transfused women have reported reduced breastfeeding rates at discharge compared to non-transfused women [59]. More research is required to investigate the impact of interventions for PPA on breastfeeding, and to guide evidence-based discussions between women and clinicians on the optimal PPA interventions to support breastfeeding.

An important finding from this systematic review was that only randomized trials of IV-iron compared to oral iron for PPA were available for meta-analysis, reflecting a scarcity of trials on RBC-T as a PPA intervention. This is concerning, given RBC-T is the traditional treatment for more severe PPA [60–62].

In contrast to patient blood management strategies which recommend IV-iron as an alternative to RBC-T, to minimize the use of RBC-Ts for stable women with PPA [10, 11, 63], our recent observational study [62] found RBC-T is often used in combination with IV-iron for PPA. The high usage of IV-iron (21–46%) in the RBC-T arms of a recent study in this review [50] may also reflect this change in treatment approach for PPA, where IV-iron is given alongside RBC-T to replenish iron-stores. It is noteworthy that only one of three studies with RBC-T as an intervention reported ferritin concentration, given adequate iron stores are essential for erythropoiesis and longer-term recovery from PPA. Current evidence comparing RBC-T to IV-iron to guide management of the very common clinical scenario of PPA is limited to one small pilot trial [49] included in this review, and a recent quasi-experimental study [64] which found IV-iron is as effective as RBC-T at improving Hb and ferritin levels at 6 weeks in stable women with PPA.

This systematic review supports findings from other reviews that IV-iron is superior to oral iron at increasing hemoglobin and ferritin concentrations for women with PPA [13, 14], but extends our knowledge by including hemoglobin and ferritin outcome data up to 12 weeks postpartum. Longer-term hematological outcomes are likely to be important for maternal and newborn wellbeing, as well as for more accurate calculation of the dose–response time of oral and IV-iron to correct anemia. Furthermore, longer-term ferritin concentrations are important when assessing the impact of IV-iron on iron stores, as there are short-term elevations in ferritin as an inflammatory marker in the immediate postpartum period, also due to the transient increase in markers of oxidative stress seen in response to IV-iron administration [65–67].

Our findings support previous findings that IV-iron is associated with significantly fewer adverse effects than oral iron, due to the high incidence of gastrointestinal side effects associated with oral iron [3, 13, 14]. High doses of oral iron are now recognized as being associated with oxidative stress and hepcidin-mediated inflammatory responses within the gut mucosa, resulting in side effects and reduced iron absorption [68, 69]. It is likely that the high oral iron dosing seen in the comparative studies of IV-iron vs. oral iron contributed to high frequencies of gastrointestinal side effects and variable compliance rates.

Reporting of IV-iron injection site reactions was difficult to interpret due to inconsistent terminology. It was unclear whether ‘extravasation’ reported in some studies resulted in skin discoloration. IV-iron site discoloration is an important outcome with potential long-term consequences and reported in only two studies [38, 49]. This may be a more common adverse event than reported in clinical trials, manufacturers’ information, and by regulatory authorities and warrants further investigation. There were no serious drug-related adverse reactions in any of the included studies.

The main strength of this review was the thorough literature search, and publication bias was not observed. A further strength of this review was that this is the first systematic review to include RBC-T alongside IV-iron as a treatment for PPA and examine the impact of treatments for PPA on both woman-centered and hematological outcomes. Other systematic reviews have [3] focused only on iron therapy [13, 14], or have not included hematological outcomes [3].

Our findings on hematological outcomes for IV-iron and oral iron are limited by the high degree of heterogeneity, which renders the evidence of low-moderate quality. The high heterogeneity was partially accounted for by post-hoc sensitivity analysis removing studies with off-protocol RBC-T in the IV-iron and oral iron groups and by pre-specified subgroup analyses of baseline Hb concentration, and high/low dose of IV-iron. However, it was challenging to account for oral iron dosing because mean doses and compliance were poorly reported, and the per protocol oral iron dosing range between studies was wide. The methodological quality of the majority of studies was not high, with few having a low risk of bias in most domains.

Our systematic review and meta-analysis examined the evidence for IV-iron and RBC-T when compared with each other, oral iron, or placebo for the treatment of PPA. We found high heterogeneity with various approaches to dosing of iron therapy for the treatment of PPA. Of the few trials on treatments for PPA that report fatigue outcomes, the quality of the evidence is low, inconsistent, and inconclusive. For all outcomes, the evidence for RBC-T is very limited. This systematic review has identified knowledge gaps in the comparison of RBC-T with IV-iron for PPA for fatigue, hematological, depression, and breastfeeding outcomes that can be used to guide future research and clinical practice.

### Supplementary Information

**Additional file 1: Figure S1.** Forest Plot of sensitivity analysis excluding off-protocol RBC-T for comparison of IV-iron vs. oral iron: Hb concentration longest follow-up (g/dL).**Figure S2.** Forest Plot of subgroup analysis of high and low dose IV-iron for comparison of IV-iron vs. oral iron Hb concentration (g/dL) longest follow-up. **Figure S3.** Forest Plot of baseline Hb concentration subgroup for comparison of IV-iron vs. oral iron Hb concentration (g/dL) longest follow-up. **Figure S4.** Forest Plot of baseline Hb subgroup and sensitivity analysis (less studies with RBC-T) for comparison of IV-iron vs. oral iron Hb concentration (g/dL) longest follow-up. **Figure S5.** Funnel Plot for comparison of IV iron vs. oral iron: Hb concentration at longest follow-up (g/dL). **Figure S6.** Forest Plot of total drug-related adverse effects for comparison of IV-iron vs. oral iron. **Figure S7.** Forest Plot of all gastrointestinal disorders for comparison of IV-iron vs. oral iron. **Figure S8.** Forest Plot of gastrointestinal disorders combined for comparison of IV-iron vs. oral iron. **Figure S9.** Forest Plot of generalized (systemic) adverse effects for comparison of IV-iron vs. oral iron. **Figure S10.** Forest Plot of all injection site disorders for comparison of IV-iron vs. oral. **Figure S11.** Forest Plot of biochemical outcomes for comparison of IV-iron vs. oral iron. **Figure S12.** Forest Plot of hypophosphataemia for comparison of IV-iron vs. oral iron. **Appendix 1. **MEDLINE (Ovid)

**Additional file 2: Appendix 2. **PRISMA 2020 Checklist 

## Data Availability

The datasets used and/or analyzed during the current study are available from the corresponding author on reasonable request.

## Abbreviations

EPDS: Edinburgh Postnatal Depression Score
GRADE: Grading of Recommendations, Assessment, Development and Evaluation
Hb: Hemoglobin
IV-iron: Intravenous iron
MD: Mean difference
MFI: Multidimensional Fatigue Inventory
PPA: Postpartum anemia
PRISMA: Preferred Reporting Items for Systematic Reviews and Meta-Analysis
PROSPERO: Prospective Register of Systematic Reviews
RR: Risk ratio
RBC-T: Red blood cell transfusion
SF-36: 36-Item Short-Form Health Survey
SMD: Standardised mean difference
