# Supplementary material for: Intravenous iron versus blood transfusion for postpartum anemia: a systematic review and meta-analysis
Source: Syst Rev. 2024 Jan 2;13:9. doi: 10.1186/s13643-023-02400-4 (PMC10759729; doi:10.1186/s13643-023-02400-4)
Supplement: Supplementary file 1 — Additional file 1: Figure S1. Forest Plot of sensitivity analysis excluding off-protocol RBC-T for comparison of IV-iron vs. oral iron: Hb concentration longest follow-up (g/dL).Figure S2. Forest Plot of subgroup analysis of high and low dose IV-iron for comparison of IV-iron vs. oral iron Hb concentration (g/dL) longest follow-up. Figure S3. Forest Plot of baseline Hb concentration subgroup for comparison of IV-iron vs. oral iron Hb concentration (g/dL) longest follow-up. Figure S4. Forest Plot of baseline Hb subgroup and sensitivity analysis (less studies with RBC-T) for comparison of IV-iron vs. oral iron Hb concentration (g/dL) longest follow-up. Figure S5. Funnel Plot for comparison of IV iron vs. oral iron: Hb concentration at longest follow-up (g/dL). Figure S6. Forest Plot of total drug-related adverse effects for comparison of IV-iron vs. oral iron. Figure S7. Forest Plot of all gastrointestinal disorders for comparison of IV-iron vs. oral iron. Figure S8. Forest Plot of gastrointestinal disorders combined for comparison of IV-iron vs. oral iron. Figure S9. Forest Plot of generalized (systemic) adverse effects for comparison of IV-iron vs. oral iron. Figure S10. Forest Plot of all injection site disorders for comparison of IV-iron vs. oral. Figure S11. Forest Plot of biochemical outcomes for comparison of IV-iron vs. oral iron. Figure S12. Forest Plot of hypophosphataemia for comparison of IV-iron vs. oral iron. Appendix 1. MEDLINE (Ovid) [file 13643_2023_2400_MOESM1_ESM.docx]

## Additional tables and figures for Systematic Review on Interventions for Postpartum Anaemia

**Additional Figure 1: Forest Plot of sensitivity analysis excluding off-protocol RBC-T for comparison of IV-iron vs. oral iron: Hb concentration longest follow-up (g/dL)**

**
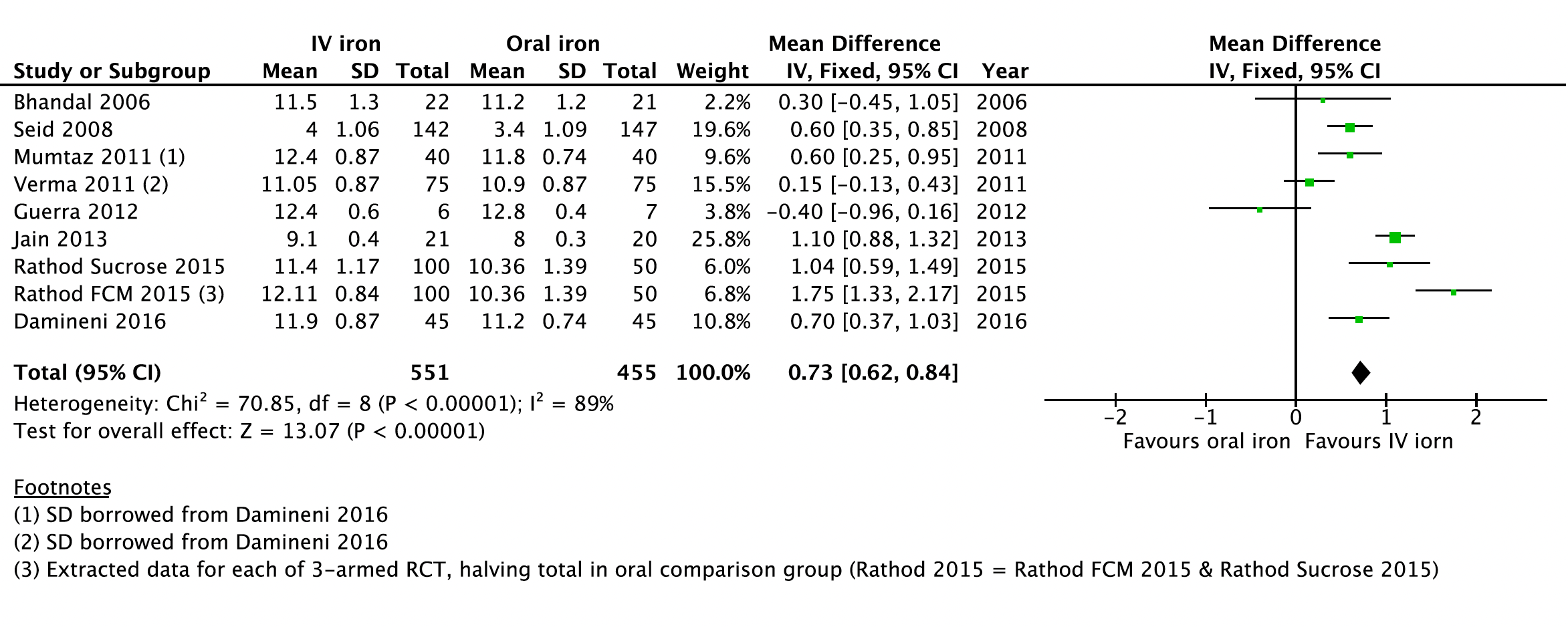
**

**Additional Figure 2: Forest Plot of subgroup analysis of high and low dose IV-iron for comparison of IV-iron vs. oral iron Hb concentration (g/dL) longest follow-up**

**
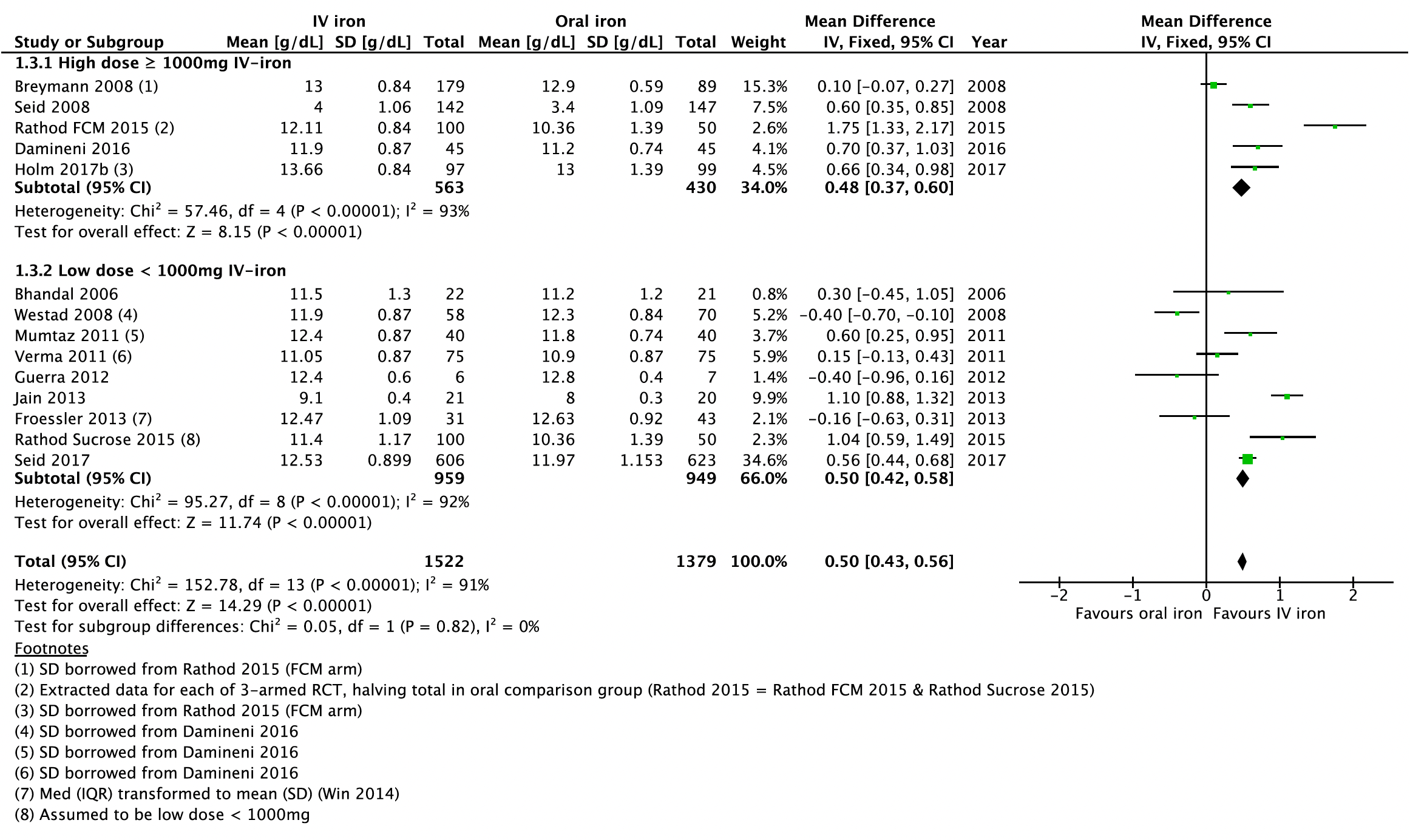
**

**Additional Figure 3: Forest Plot of baseline Hb concentration subgroup for comparison of IV-iron vs. oral iron Hb concentration (g/dL) longest follow-up**

**
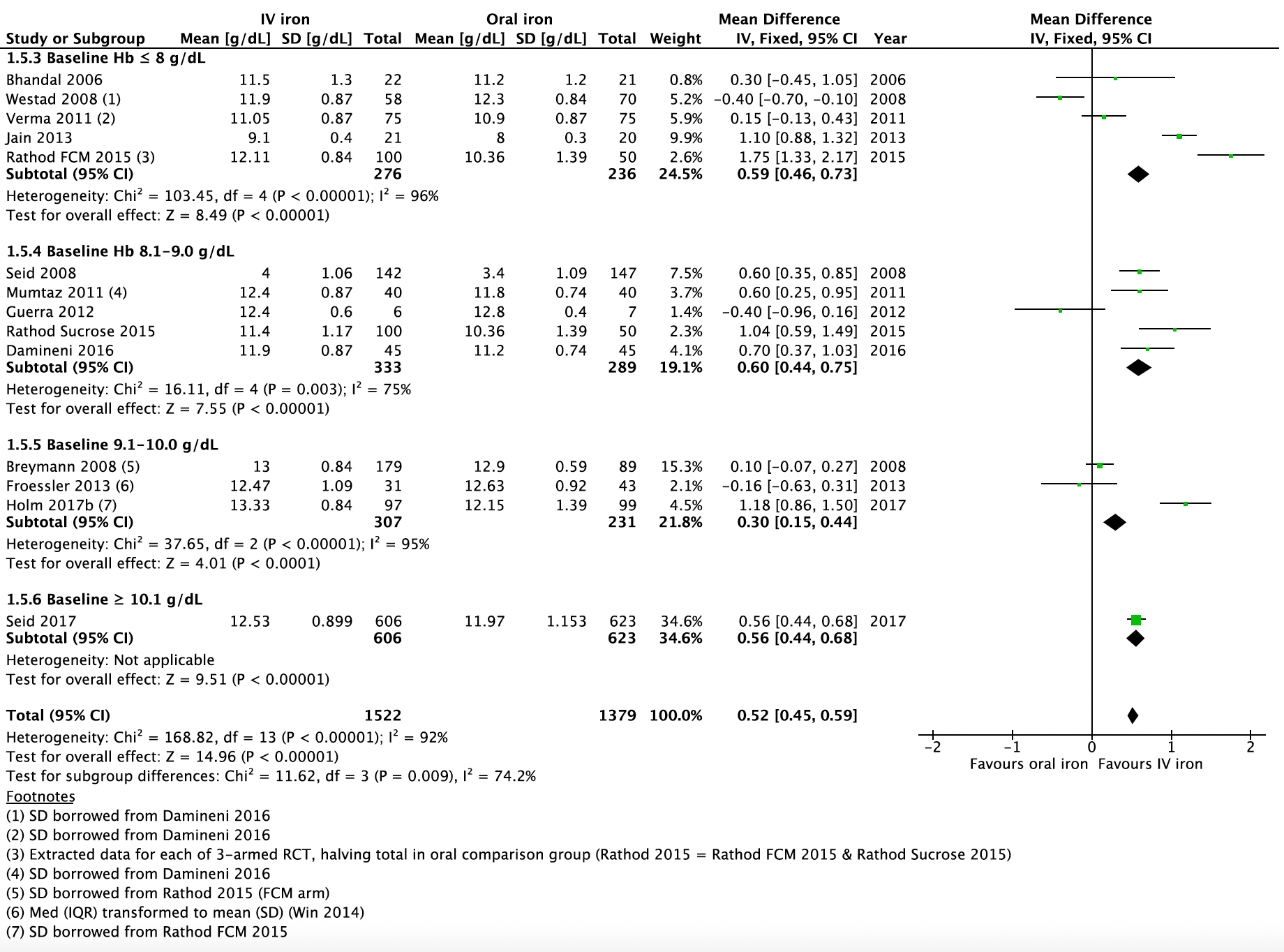
**

**Additional File Figure 4: Forest Plot of baseline Hb subgroup and sensitivity analysis (less studies with RBC-T) for comparison of IV-iron vs. oral iron Hb concentration (g/dL) longest follow-up**

**
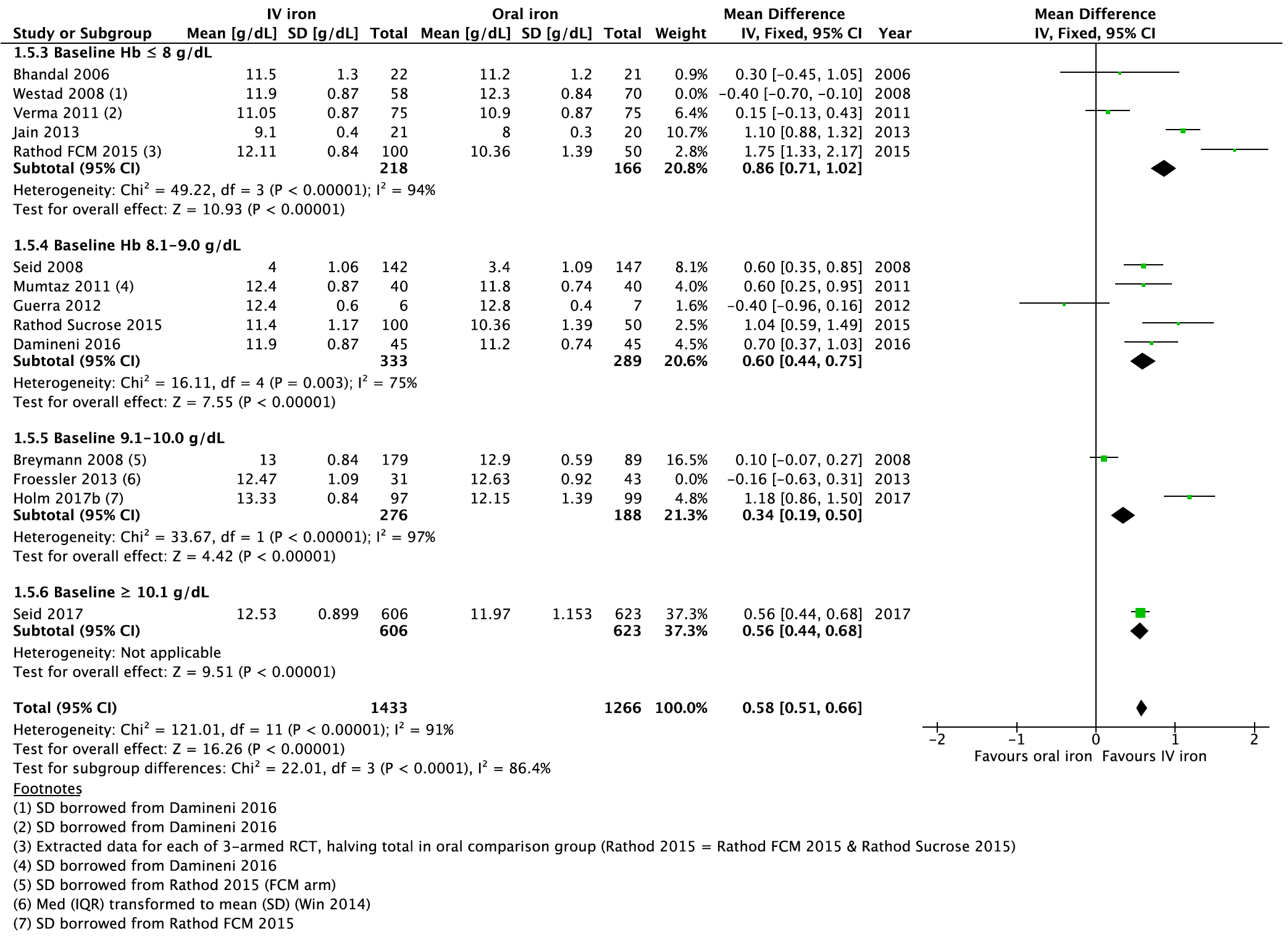
**

**Additional Figure 5: Funnel Plot for comparison of IV iron vs. oral iron: Hb concentration at longest follow-up (g/dL)**


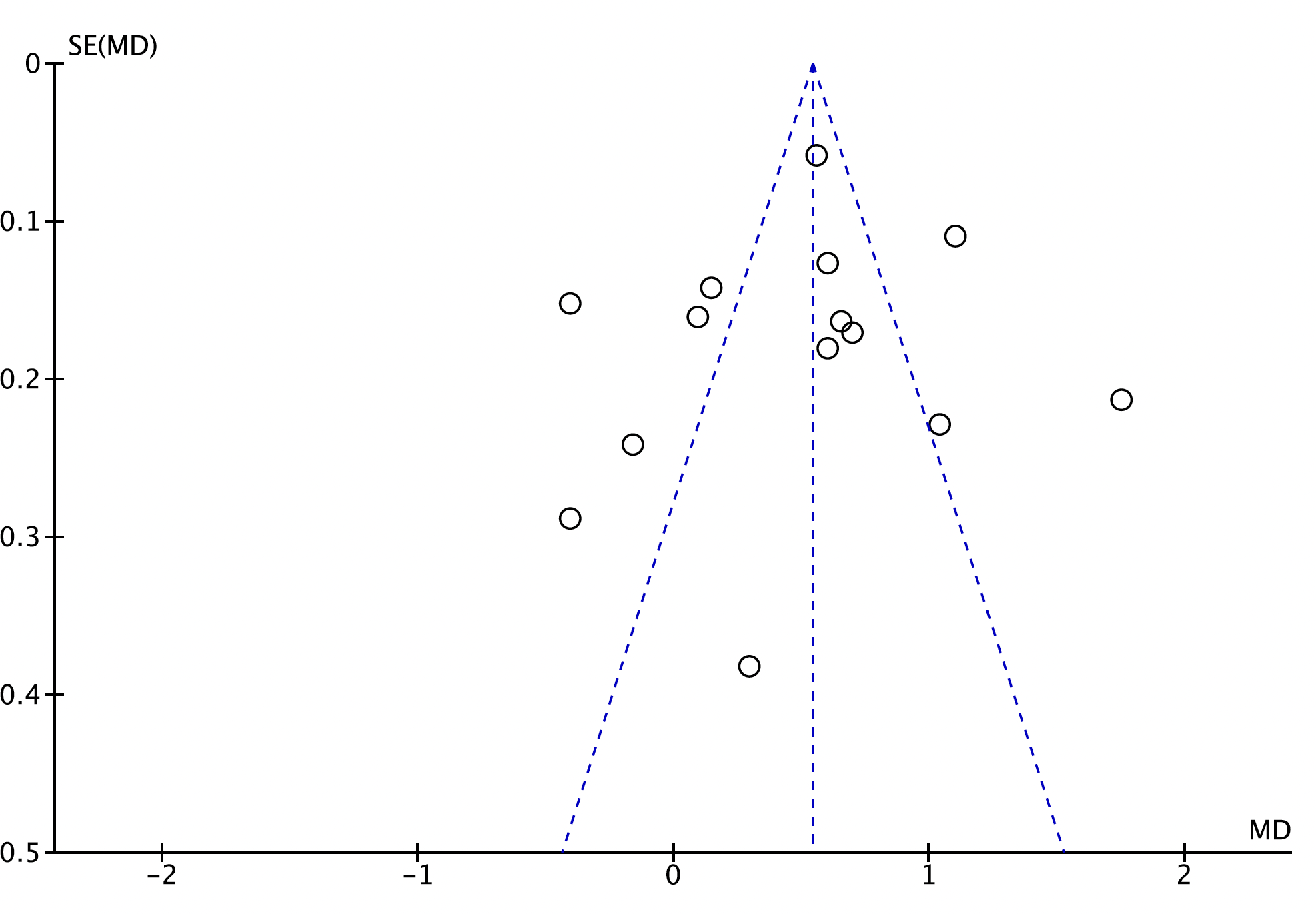


**Additional File Figure 6: Forest Plot of total drug-related adverse effects for comparison of IV-iron vs. oral iron**


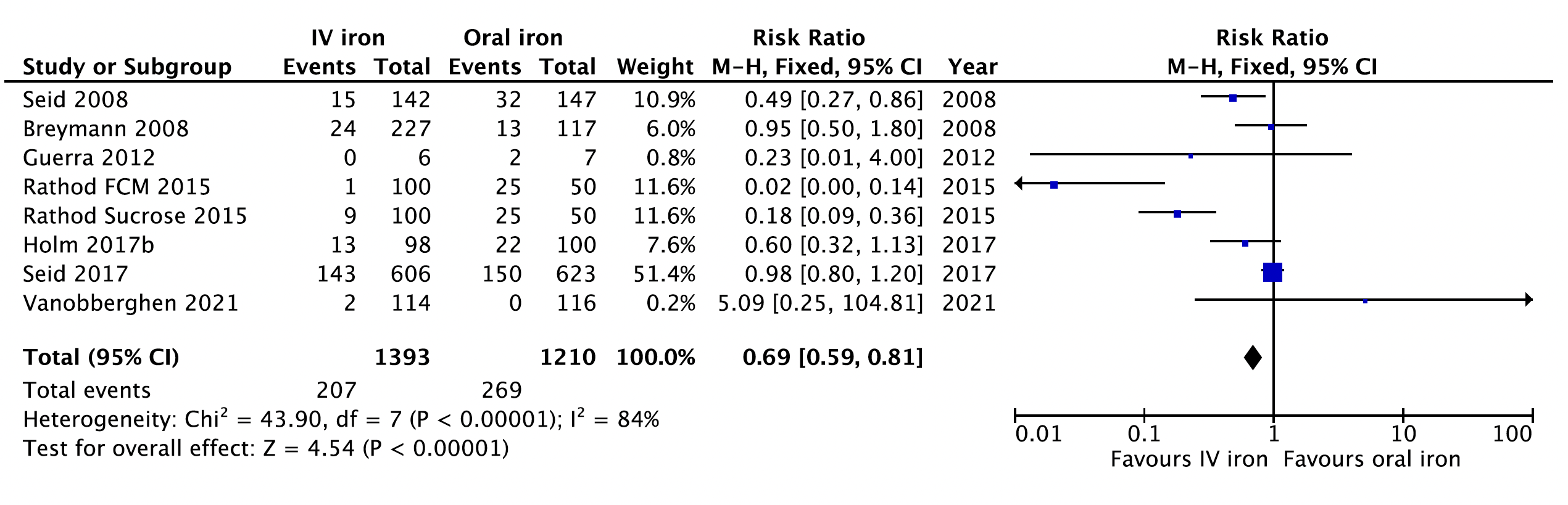


**Additional Figure 7: Forest Plot of all gastrointestinal disorders for comparison of IV-iron vs. oral iron**

**
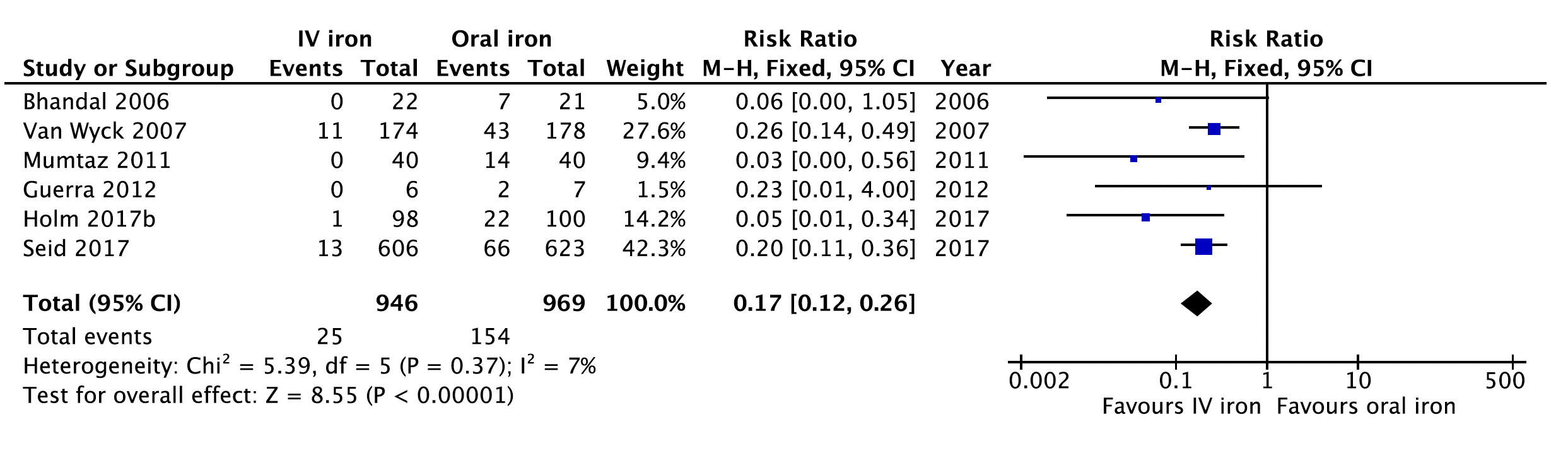
**

**Additional Figure 8: Forest Plot of gastrointestinal disorders combined for comparison of IV-iron vs. oral iron**

**
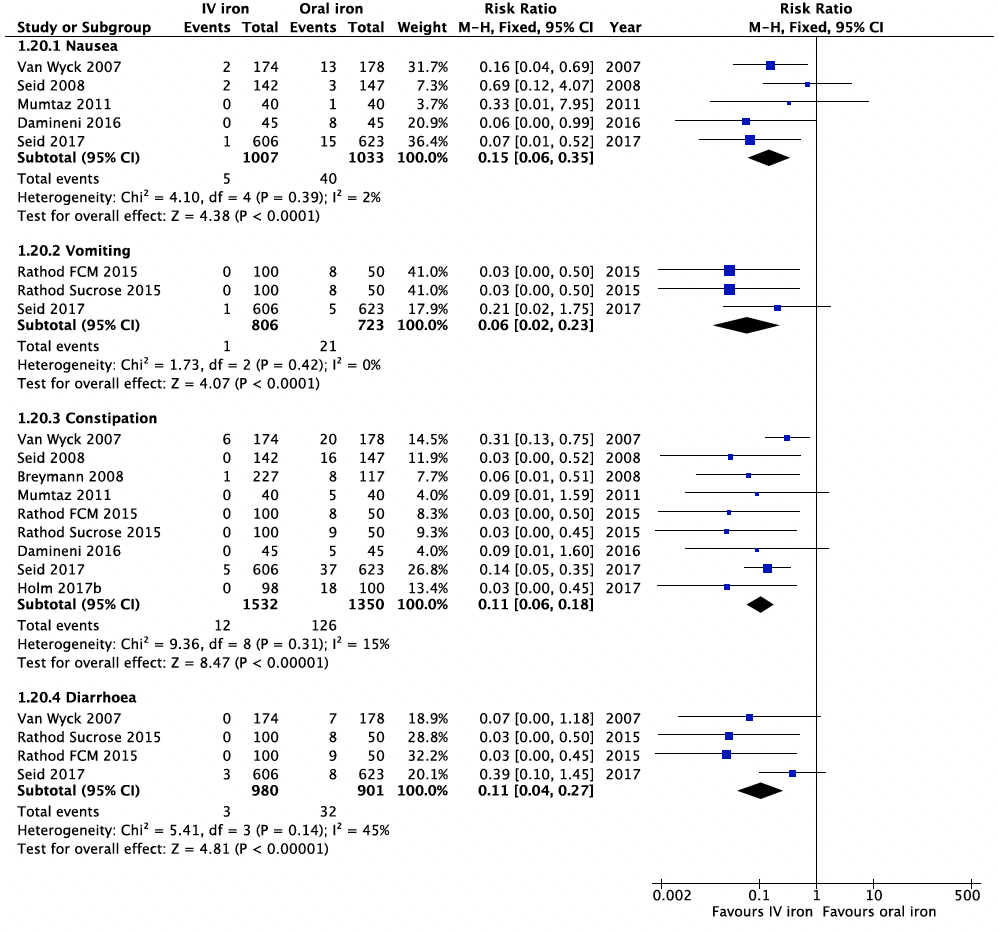
**

**Additional Figure 9: Forest Plot of generalized (systemic) adverse effects for comparison of IV-iron vs. oral iron**

**
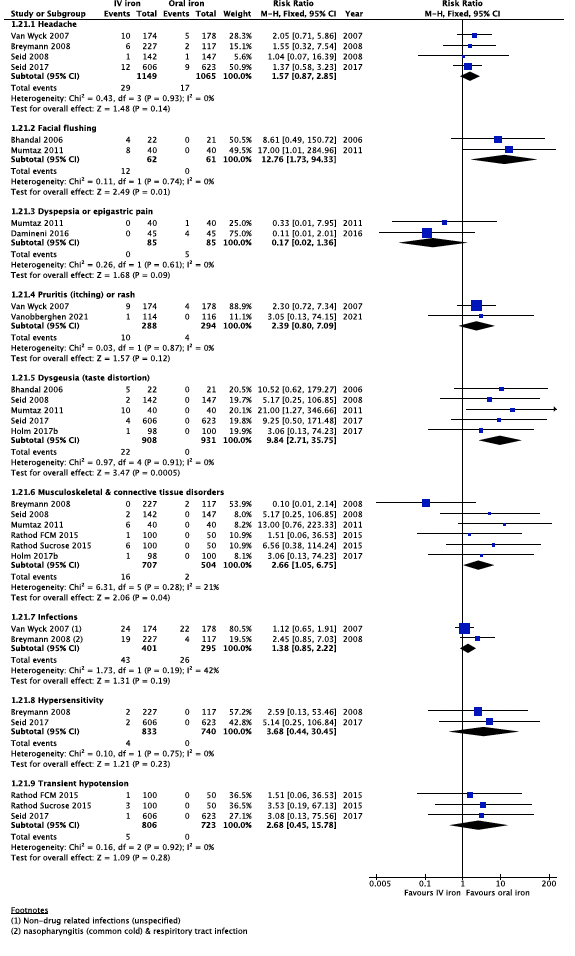
**

**Additional Figure 10: Forest Plot of all injection site disorders for comparison of IV-iron vs. oral**

**
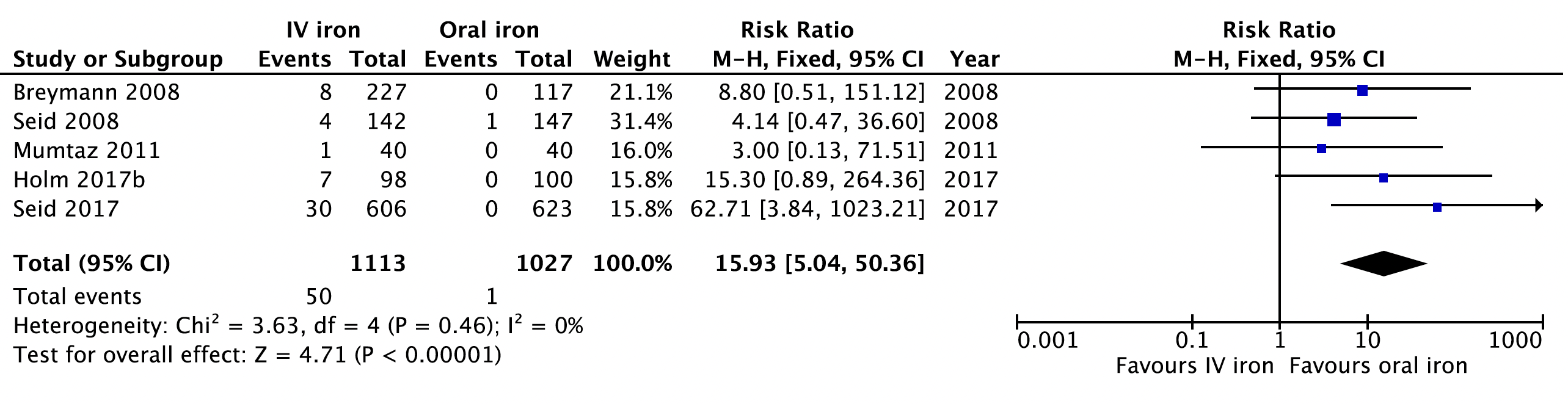
**

**Additional Figure 11: Forest Plot of biochemical outcomes for comparison of IV-iron vs. oral iron**

**
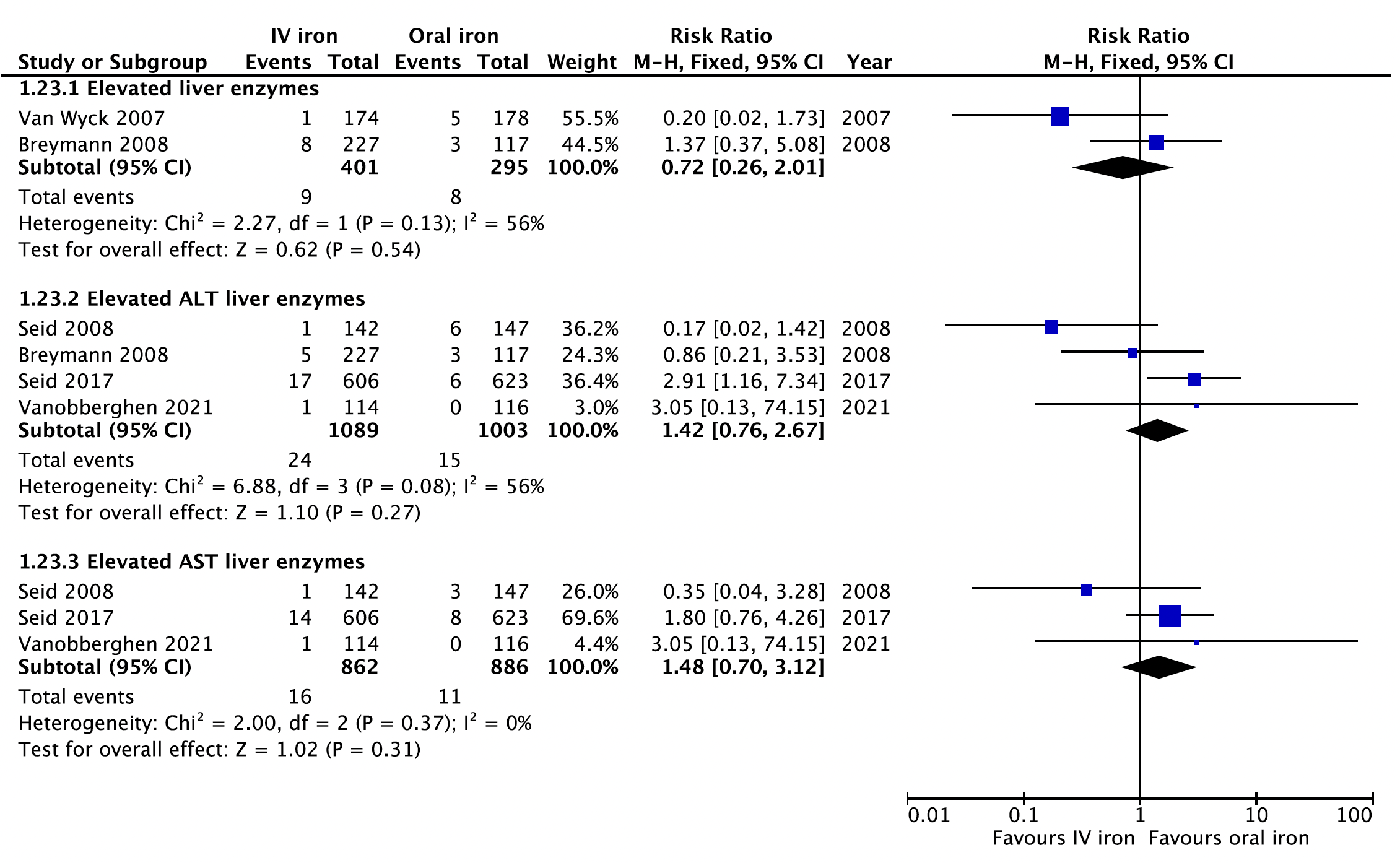
**

**Additional Figure 12: Forest Plot of hypophosphataemia for comparison of IV-iron vs. oral iron**

**
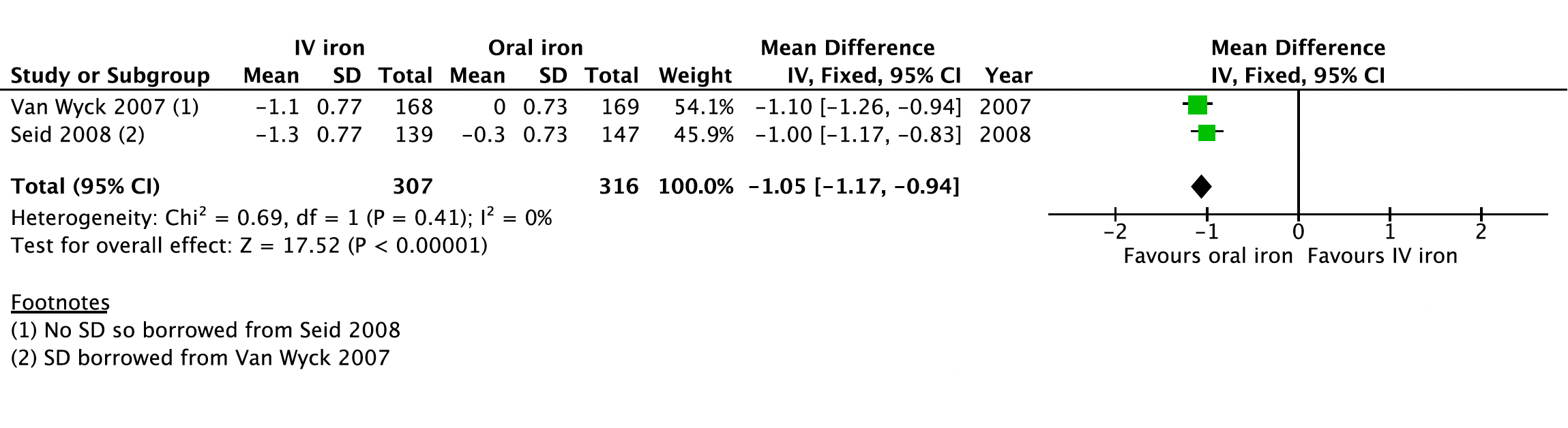
**

**Additional File Appendix 2.**

**MEDLINE (Ovid)**

1. Postpartum Period/
2. puerperal disorders/
3. (postpartum or post partum or postnatal* or puerper*).ti,ab.
4. 1 or 2 or 3
5. Anemia/
6. Anemia, Iron-deficiency/
7. Postpartum Hemorrhage/
8. (anaemia or anemia or anaemic or anemic or postpartum hemorrhage or haemorrhage or iron-deficien*).ti,ab.
9. 5 or 6 or 7 or 8
10. 4 and 9
11. Iron/
12. Ferric Compounds/
13. Ferrous Compounds/
14. Hematinics/ or iron-dextran complex/
15. (iron or ferric compounds or ferrous or carboxymaltose or ferinject or iron-sucrose or venofer or isomaltoside or monofer or iron polymaltose or maltofer).ti,ab.
16. Erythrocyte Transfusion/
17. (erythrocyte transfusion or red blood cells or blood transfusion or allogenic).ti,ab.
18. 11 or 12 or 13 or 14 or 15 or 16 or 17
19. Administration, intravenous/ or infusion, intravenous/ or injections, intravenous/
20. (intravenous administration or infusion* or injection*).mp.
21. 19 or 20
22. 18 and 21
23. Randomized Controlled Trials as Topic/
24. Controlled clinical trial.mp
25. (randomi?ed controlled trial or clinical trial or random* or placebo).ti,ab.
26. 23 or 24 or 25
27. 10 and 22 and 26
